# Chronic p27^Kip1^ Induction by Dexamethasone Causes Senescence Phenotype and Permanent Cell Cycle Blockade in Lung Adenocarcinoma Cells Over-expressing Glucocorticoid Receptor

**DOI:** 10.1038/s41598-018-34475-8

**Published:** 2018-10-30

**Authors:** Mugdha Patki, Thomas McFall, Rayna Rosati, Yanfang Huang, Agnes Malysa, Lisa Polin, Abigail Fielder, Mike R. Wilson, Fulvio Lonardo, Jessica Back, Jing Li, Larry H. Matherly, Gerold Bepler, Manohar Ratnam

**Affiliations:** Barbara Ann Karmanos Cancer Institute and Department of Oncology, Wayne State University, Detroit, MI 48201-2013 USA

## Abstract

Dexamethasone (Dex), co-administered to lung adenocarcinoma patients with pemetrexed chemotherapy, protects against pemetrexed cytotoxicity by inducing reversible G1 arrest, reflected by the effect of Dex on FLT-PET images of patient tumors. However, perioperative Dex treatment increases survival but the mechanism is unknown. In cells with glucocorticoid receptor-α (GR) expression corresponding to higher clinical tumor levels, Dex-induced growth arrest was followed by marked cell expansion, beta-galactosidase expression and Ki67 negativity, despite variable p53 and K-RAS status. Dex induced a transient early surge in p21^Cip1^. However, a progressive, irreversible loss of clonogenic growth, whose time of onset was dependent on GR level and Dex dose, was independent of p21^Cip1^and caused by gradual accumulation of p27^Kip1^ due to transcriptional activation of p27^Kip1^ by Dex. This effect was independent of canonical pathways of senescence or p27^Kip1^ regulation. The *in vitro* observations were reflected by growth suppression and P27^Kip1^ induction in GR-overexpressing tumor xenografts compared with isogenic low-GR tumors. Extended Dex treatment induces irreversible cell cycle blockade and a senescence phenotype through chronic activation of the p27^Kip1^ gene in GR overexpressing lung tumor cell populations and hence could improve outcome of surgery/pemetrexed chemotherapy and sensitize tumors to immunotherapy.

## Introduction

Lung adenocarcinoma (non-squamous non-small cell lung cancer) comprises over half of all lung cancers with over 100,000 newly diagnosed cases each year. The majority have advanced disease with estimated 5-year survival of 4.5% and median overall survival of 18 months. The mainstay chemotherapy drug pemetrexed, in combination with a platinum agent, showed median overall survival of 12.6 months in the first phase 3 trial^1^ and is used in first-line and maintenance therapy^2^ and almost always used following immunotherapy or targeted therapies. About 23–28% of patients express relatively high levels of PD-L1 and qualify for immunotherapy with a PD-1/PD-L1 inhibitor which in a recent phase 3 trial gave an increased benefit compared to chemotherapy in terms of median progression-free survival (10.3 months vs 6.7 months) and overall survival at 6 months (80.2% vs 72.4%)^3^. Targeted therapies including protein tyrosine kinase inhibitors, ALK inhibitors and angiogenesis inhibitors extend survival in smaller cohorts^4^. There is a pressing need to achieve better treatment outcomes in lung adenocarcinoma.

In pemetrexed-based chemotherapy, dexamethasone (Dex) is co-administered to alleviate drug-induced severe and painful (grades 3 and 4) skin rash^5,6^. Using a panel of lung adenocarcinoma cell lines, we have previously shown that Dex could reversibly arrest the tumor cells in the G1 phase of the cell cycle and that the cells would then slowly resume proliferation after Dex withdrawal^7^. In the Dex-responsive cell lines, cytotoxicity of pemetrexed was thus abrogated by Dex, irrespective of expression/mutation status of p53 or K-RAS^7^. Correlative and gain-of-function evidence pointed to tumor glucocorticoid receptor type α (GR) expression status as the principal determinant of variability in this “Dex response” among the cell line models. The GR status-dependent reversible growth arrest by Dex was also evident in isogenic GR-high vs. GR-low cells^7^. The clinical relevance of this effect was supported by a retrospective study of patients that received pemetrexed chemotherapy^8^. The clinical relevance was also confirmed using positron emission tomography (PET) imaging in lung adenocarcinoma patients that measured the effect of 24 h of Dex treatment on tumor retention of the proliferation tracer 3′-fluoro-3′-deoxy-thymidine (FLT). In one of four patients, Dex caused decline in the FLT-PET signal in all tumor lesions and in two patients, the declines were variable among multiple tumor lesions^9^. Thus in a cohort of patients with tumor lesions expressing relatively high levels of GR, Dex may attenuate the anti-tumor effects of the very chemotherapy whose side effects it is being used to alleviate.

A large retrospective study showed peri-operative administration of Dex increased survival in non-small cell lung cancer and there is currently an open prospective trial to evaluate this effect^10^. It is speculated that this could be related to post-operative stress and associated immune effects but direct effects of Dex on the tumors have not been examined in this context. Knowledge of the underlying mechanisms, is needed to optimize treatment and stratify patients for this survival benefit. GR exerts a broad range of transcriptional effects that are cell type specific^11^. Although long-term systemic glucocorticoid therapy is used for its anti-inflammatory effects^12^, reported cellular effects of glucocorticoids are typically limited to short-term (~24 h) treatments. In certain malignant cell types, this may immediately result in either reversible growth inhibition or apoptosis^7,13,14^. In some instances, even brief exposure to glucocorticoids may induce lasting cellular effects, such as epigenetic reprogramming observed in primary embryonic rat neural stem cells^15^. Additionally, in patients who received local injection of glucocorticoid for rotator cuff tendinopathy, tendon biopsies showed a lasting (up to 7 weeks) increase in acetylated p53 in association with senescence markers^16^. It is also noteworthy that although GR is the principal mediator of glucocorticoid actions, there is very little information on GR expression levels in relation to the nature of the cellular response to the hormone. In light of our previous studies of lung adenocarcinoma cells, it was of interest to investigate whether extended exposures of Dex-sensitive (GR overexpressing) lung adenocarcinoma cells to Dex may render them unable to recover from cell cycle arrest following Dex withdrawal. The theoretical basis for this possibility is that steady state arrest in G1 phase is not sustainable, and mechanisms of G1 arrest overlap pathways leading to quiescence (reversible), senescence (reversible or irreversible) or cell death^17,18^. As Dex is relatively innocuous even when administered for several weeks, it was desirable to explore pre-clinically, long-term treatment with Dex as potentially an additional supporting intervention for a selected cohort of lung adenocarcinoma patients.

## Methods

### Cell culture and Reagents

Lung adenocarcinoma cell lines A549, H292 and H1299 were grown in RPMI 1640 medium supplemented with 10% fetal bovine serum (FBS), 100 units/ml penicillin, 100 μg/ml streptomycin and 2 mM glutamine (Thermo Fisher Scientific, Waltham, MA). Two clones of stable recombinant H1299 cells overexpressing GRα (H1299GRα Clone 2 and H1299GRα Clone 4 cells) were produced, as described previously^7^. A549 and H292 cells express wtp53 whereas H1299 cells are p53null. A549 cells harbor a G12S mutation in K-RAS whereas H292 and H1299 cells express wtRAS. The origin, growth conditions and responsiveness to selected agents of the cell lines used in this study have been previously described^19^. All cell lines had been authenticated by SNP profiling within less than 12 months of *in vitro* propagation. Dexamethasone was purchased from EMD Millipore (Billerica, MA). Crystal violet was purchased from Thermo Fisher Scientific. TaqMan probes for qPCR were either purchased from the Thermo Fisher Scientific inventory or custom synthesized by Integrated DNA Technologies (Coralville, IA). The GRα-expressing lentivirus was from GenTarget Inc. (San Diego, CA). Blasticidin and puromycin were from Thermo Fisher Scientific and Sigma-Aldrich, St. Louis, MO, respectively. p21^Cip1^ shRNA was from Sigma-Aldrich and FOXO3 (#L-003007-00-0005), FOXO4 (#L-003016-00-0005) and p27^Kip1^ siRNAs (#L-003472-00-0005) were from GE Dharmacon (Lafayette, CO). pLenti-p27^Kip1^ expression vector (#RC201661L1) was purchased from Origene, Rockville, MD. Anti-GR (#12401), p27^Kip1^ (#2552), p21^Cip1^ (#2947), FOXO3a (#75D8) and FOXO4 (#55D4) antibodies were purchased from Cell Signaling Technology, Danvers, MA. Anti-p16^INK4a^ (#ab81278) was from Abcam. Anti-α-tubulin (sc-8035) and GAPDH (sc-47724) antibodies were purchased from Santa Cruz Biotechnology, Dallas, TX.

### Gene Transduction

For lentivirus mediated overexpression or knockdown p21^Cip1^ shRNA, non-target control shRNA, pLenti-p27^Kip1^ expression plasmid and control expression vector were packaged in 293FT cells using lentiviral packaging plasmids. The virus-containing supernatant was harvested 48 h and 72 h after transfection, filtered and stored at −80 °C until the time of infection. 24 h before infection, cells were plated in 6-well plates. The next day, cells were infected with appropriate control and target lentivirus with polybrene (8 μg/mL) for a duration of 5 h followed by a similar second lentiviral infection for an additional 5 h. The media was then replaced with fresh phenol red free medium containing 10% FBS. Recombinant cells were selected with appropriate mammalian selection marker.

### Transfection of siRNA

Cells were plated at 20% confluence in 6-well plates. Cells were transfected with either control siRNA, or target siRNA at a concentration of 100 pmol/mL with 5 μl of Dharmafect 1 (GE Dharmacon) according to the vendor’s protocol. After 48 h of knockdown, fresh media containing respective treatments were added to the cells. Cells were harvested for analysis at the appropriate time points.

### RNA extraction and real time PCR

To measure gene expression, mRNA was quantified by real time reverse transcription PCR. Total RNA from cells was isolated using PureLink RNA extraction kit (Thermo Fisher Scientific) according to the manufacturer’s protocol. Reverse transcription was performed using 500 ng of total RNA and High-Capacity cDNA Archive kit (Thermo Fisher Scientific), according to the vendor’s protocol. cDNA was measured by quantitative real-time PCR using the StepOnePlus Real-Time PCR System (Thermo Fisher Scientific) and TaqMan Fast Universal PCR Master Mix (Thermo Fisher Scientific). All of the mRNA measurements were carried out using biological triplicate samples and C_T_ values were normalized to those of GAPDH.

### Western Blot Analysis

Cell lysates were generated using RIPA buffer (150 mM NaCl, 1% NP-40, 0.5% sodium deoxycholate, 0.1% SDS, 50 mM Tris of pH 8.0) containing Halt protease inhibitor cocktail (Thermo Fisher Scientific) and incubated on ice for 1 h. Total protein concentration was determined by the Bradford assay (Bio-Rad Laboratories, Hercules, CA). Protein samples were resolved on 8% SDS-polyacrylamide gels and electrophoretically transferred to PVDF membranes (Millipore Corporation, Bedford, MA). The blots were probed with the appropriate primary antibody and the appropriate horse radish peroxidase conjugated secondary antibody. The protein bands were visualized using enhanced chemiluminescence reagents (Hyglo Quick Spray, Denville Scientific).

### Colony formation assay

After appropriate treatment, cells were trypsinized and 2000 cells per well were plated in triplicate in 6-well plates in phenol-red-free medium supplemented with FBS. After allowing one week for colony formation, cells were fixed with ice cold methanol and stained with crystal violet. Images were obtained using the Oxford Optronix GelCount colony counter. Colonies were counted using the Oxford Optronix GelCount Software Version 1.1.2.0 (Oxford Optronix Ltd., Abingdon, UK). The colony formation assays were repeated at least three times.

### Cell Proliferation assay

Cells were seeded in 6-well plates and treated in triplicate. At the appropriate time point, cells were trypsinized and 10 µl of the cell suspension was mixed with 10 µl of trypan blue solution (Thermo Fisher Scientific). The cells were loaded on a hemacytometer and counted under a microscope. Cell viability was calculated and the viable cell counts were monitored for 7 days.

### Apoptosis assay

Cells were trypsinized and washed with cold phosphate-buffered saline. The cells were suspended in 1x binding buffer and stained with either PE Annexin V or APC Annexin V using an apoptosis detection kit (BD Biosciences, San Jose, CA) per the manufacturer’s protocol. Apoptotic cells were quantified using the BD LSR II analyzer (BD Biosciences) at the Microscopy, Imaging and Cytometry Resources (MICR) Core at Karmanos Cancer Institute. The data was analyzed using FloJo software to calculate the percentage of annexin V+ cells. Cisplatin (20 µM) treated cells were used as a positive control for apoptotic annexin V+ cells. Each treatment was analyzed in biological triplicate.

### Measurement of cell size

Cells were seeded in 6-well plates and trypsinized following the appropriate treatment. The cells were washed with phosphate-buffered saline and suspended at a concentration of 1 × 10^6^ cells per 100 µl of PBS. Cells were analyzed using the Amnis ImageStream Mark II Imaging Flow Cytometer (EMD Millipore) at the MICR Core at Karmanos Cancer Institute. Cell images were captured at a magnification of 60x and analyzed using IDEAS 4.0 software (EMD Millipore). Gating was applied based on single cell population and cells that were focused. Areas of the cells were calculated and the data were plotted as histograms.

### Senescence associated-β-galactosidase staining

Cells were plated in 6-well plates and appropriately treated. At the end of each time point, the cells were stained using the senescence β-galactosidase staining kit (#9860, Cell Signaling Technologies, Danvers, MA) per the vendor’s procedure. After staining was completed, cell images were captured using a microscope at a magnification of 20 ×. A total of 30 images were captured from triplicate wells and representative images were chosen for presentation.

### Analysis of cell cycle distribution and Ki67 negativity

Cells were trypsinized and harvested in phenol-red free medium supplemented with FBS. Cells (1 × 10^6^) were washed and suspended in 500 ul of phosphate-buffered saline (PBS). The cells were fixed with ice-cold 100% ethanol and incubated at −20 °C overnight. The cells were pelleted and washed 2X with PBS and incubated with Alexa Fluor® 647 Mouse anti-Ki-67 antibody (Catalog No.558615, BD Biosciences) for 30 minutes at room temperature. Cells were then washed 2X with PBS and suspended in 500 ul of propidium iodide/RNase solution and incubated in the dark at room temperature for 4 hours. Cell-cycle distribution and Ki67 expression were determined using the BD LSR II analyzer (BD Biosciences) at the Microscopy, Image and Cytometry Resources Core at Karmanos Cancer Institute. The data were analyzed using ModFit software.

### GR expression analysis in lung tumor cDNA array

Patient cDNAs were purchased from Origene (Rockville, MD), including 26 non-squamous non-small cell lung cancer (NS-NSCLC) specimens (8, stage I; 5, stage II; 7, stage III; and 6, stage IV) and eight unmatched normal lung specimens. Quantitative real-time RT-PCR was performed using a Roche LightCycler 480 (Roche Diagnostics, Indianapolis, IN) with gene-specific primers and FastStart DNA Master SYBR Green I Reaction Mix (Roche Diagnostics). Transcript levels were normalized to transcript levels of β-actin.

### Tissue microarrays and immunohistochemistry

The human lung adenocarcinoma tissue microarray (TMA) (Catalog#: HLug-Ade150Sur-02) was purchased from US Biomax Inc. The tumor tissues were obtained pre-treatment and included Grades 1–3 and Stages I to III. For the mouse tumor xenograft studies, tissue sections were first stained with H&E and marked by the pathologist co-investigator (F.L) to denote tumor rich areas. Using the marked slides, tumor tissue cores (0.6 mm, 2 mm depth) were obtained using a manual tissue arrayer (Estigen, MTA-16003) in triplicate and randomly distributed on the slide using Leica paraffin tape transfer (Alphametrix, catalog#: 02-PSA). The TMAs included an orientation core (lymph node) and normal tissues controls that included kidney, brain cortex, colon, lung, liver and lymph node.

To perform immunohistochemistry (IHC), Vecta Elite ABC-HRP Rabbit IgG kit (Vector Labs, Catalog#: PK-4001) was used to stain slides for GR (Cell signaling, catalog#: 12041 S) and p27 (Abcam, catalog#: ab32034). Antigen retrieval was performed using Vector Antigen Unmasking solution (Citric Acid Based, Vector Labs, Catalog#: H-3300) with the following microwave protocol: Power 2 (5 minutes) followed by Power 3 (6-7 minutes) ending with Power 2 (5 minutes). Endogeneous peroxidases were blocked with 3% H_2_O_2_ at room temperature for 10 minutes for GR and 8 minutes for p27. Normal blocking solution (from Vecta Elite kit) was used to block slides for 1 hour at room temperature. Antibodies were incubated at the following dilutions at room temperature for 1 hour: 1:100 (GR) and 1:50 (p27). Rabbit biotinylated secondary antibody and ABC reagent were incubated for 30 minutes each at room temperature. DAB incubation times were: 24 seconds (GR), 45 seconds and (p27).

GR staining was scored by the pathologist co-author (F.L.) for staining intensity on a scale of 0 (no staining) to 3+ (strongest staining) and the extent of staining was scored in terms of percent positivity.

### Isogenic tumor xenograft models

The animal model studies were conducted in accordance with the Guide for the Care and Use of Laboratory Animals as adopted and promulgated by the U.S. National Institutes of Health, and were approved by our Institution’s Animal Care and use Committee. The antitumor activity of Dex or placebo treatments were evaluated *in vivo* in female SCID mice bearing xenografts of the parental H1299 (low GR) cells and H1299GR Clone 4 (high GR) cells. The tumors were maintained by serial transplantation in the flanks of SCID mice. For the experiment, tumor fragments (50–100 mg) were implanted in both flanks of SCID mice that weighed within 5 grams of each other, all exceeding a 17 gram minimum mouse weight for the start of treatment. To enable extended Dex administration, slow release subcutaneous Dex or placebo pellets were implanted when tumors reached 200–250 mg. SCID mice implanted with Dex (2.5 mg) or control placebo pellets were then monitored for tumor growth via caliper measurements 3×/week (Tumor volume = ½[L*W^2^]). Change in weight and behavior was also monitored daily, nutrition supplied ad libitum.

### Measurement of plasma Dex levels

Serum was separated from whole blood by centrifugation (at 4 °C, 10,000 × g for 10 minutes), and the resultant serum samples were stored at −80 °C until analysis. Serum concentrations of Dex were determined using a liquid chromatography coupled with tandem mass spectrometry (LC-MS/MS) method, as follows. 100 μl of mouse serum was extracted by adding 1 mL ethyl acetate followed by vortex-mix for 1 minute and centrifugation (at 4 °C, 10,000 g for 10 minutes). The top layer was transferred to a 1.5-ml polypropylene screw top tube, and evaporated to dryness under a stream of nitrogen in a water bath at 50 ± 2 °C. The residue was reconstituted in 100 µl of mobile phase followed by vortex-mix and centrifugation (at 4 °C, 10,000 g for 5 minutes). The supernatant was transferred to an autosampler vial, and 10 µL was injected into the LC-MS/MS system using a temperature-controlled autosampling device (set at 4 °C). LC-MS/MS analyses were performed using a Waters LC-MS/MS system consisting a AQUITY UPLC system coupled with a Waters Quattro Micromass triple quadrupole mass spectrometer (Milford, MA, USA). Chromatographic separation was achieved on a Waters ACQUITY UPLC BEH C18 column (2.1 × 50 mm, 1.7 µm) using an isocratic elution consisting of mobile phase A (10 mM ammonium acetate in methanol) and mobile phase B (10 mM ammonium acetate, pH 8) (60:40, v/v), at a flow rate of 0.3 mL/min. Dex was monitored under positive electrospray ionization mode, using the most sensitive and specific MS transition, m/z 391.5 > 361.3. The linear calibration curve was established at Dex concentration range of 5–5000 nM in mouse serum. The precision and accuracy of quality control samples were within generally acceptable criteria for bioanalytical methods.

### Statistical analysis

Statistical significance was determined using one way ANOVA followed by post hoc t-test to compare pairs of samples. The error bars in all graphs represent standard deviation of the mean.

## Results

### GR distribution is variable in clinical tumor specimens and cell line models

**Figure 1:**
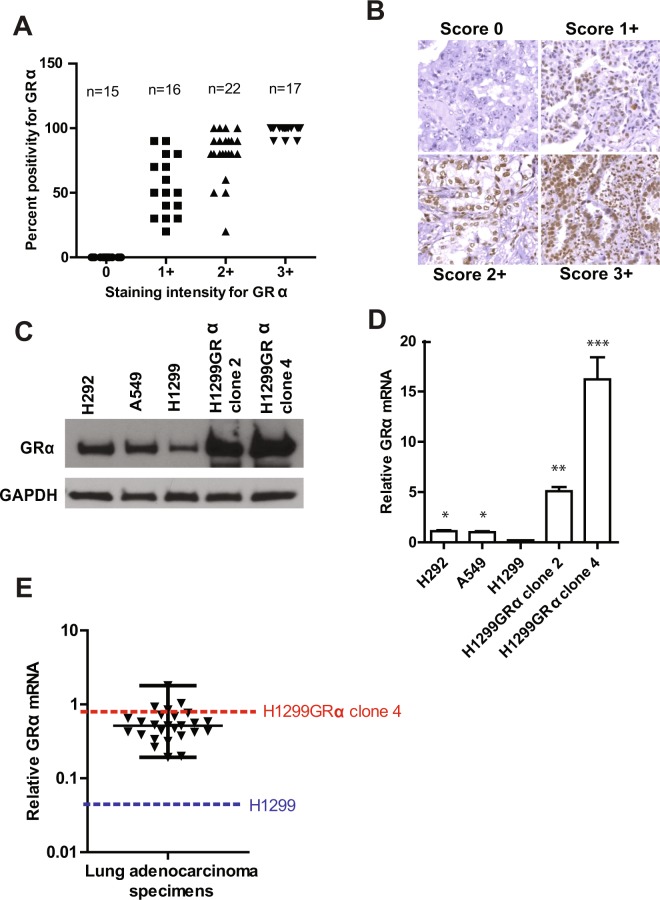
Expression of GR in clinical lung adenocarcinoma and cell line models. A lung adenocarcinoma tissue microarray was probed by immunohistochemistry for expression of GR and the expression levels were scored in the malignant cells by intensity of staining as well as percent of cells that stained positive (Panel A). Representative images showing the different intensities of staining for GR are shown (Panel B). Whole cell lysates were extracted from H292, A549, H1299, H1299GR Clone 2 and H1299GR Clone 4 cells and GR expression was visualized by western blot using GAPDH as the loading control (Panel C). RNA was also extracted from the same cell lines and the levels of GR mRNA were measured by real time RT-PCR using TaqMan probes and plotted on a linear scale (Panel D). GR mRNA was measured by quantitative RT-PCR using SYBR Green in a cDNA microarray of clinical lung adenocarcinoma tumors; the relative levels of GR mRNA in H1299 and H1299GR Clone 4 cells were simultaneously measured using SYBR Green and the data plotted on a log scale (dashed lines) (Panel E). Panel D: *P, 0.002; **P, 0.001; ***P, 0.003.

Evaluation of a lung adenocarcinoma tissue microarray stained for GR on an intensity scale ranging from 0 to 3+ showed heterogeneity in both staining intensity and the percentage of tumor cells that were positive (Fig. 1A). However, 50 percent of sections (35 of 70) showed relatively high levels (2+ and 3+) of GR in >70 percent of the tumor cells (Fig. 1A). Representative images showing the different intensities of staining for GR are shown in Fig. 1B. The lung adenocarcinoma cell line models chosen for this study included cells expressing low (H1299 cells) or moderate (A549 and H292 cells) levels of GR, or recombinant H1299 cells expressing relatively high levels of GR (H1299GR Clone 2 and H1299 GR Clone 4), as determined by western blots (Fig. 1C). The relative GR protein levels in these cell lines were reflected by their relative GR mRNA levels (Fig. 1D). Measurement of GR mRNA in a human lung adenocarcinoma cDNA array showed that 30 percent of samples (7 of 26) had GR mRNA levels that were equal to or greater than that in the recombinant H1299GR Clone 4 cells (Fig. 1E). Therefore, the cell line models cover the range of GR expression in primary lung adenocarcinoma specimens.

### Dex induces cell growth arrest and progressive loss of proliferation potential in a GR status- and Dex dose-dependent manner

**Figure 2:**
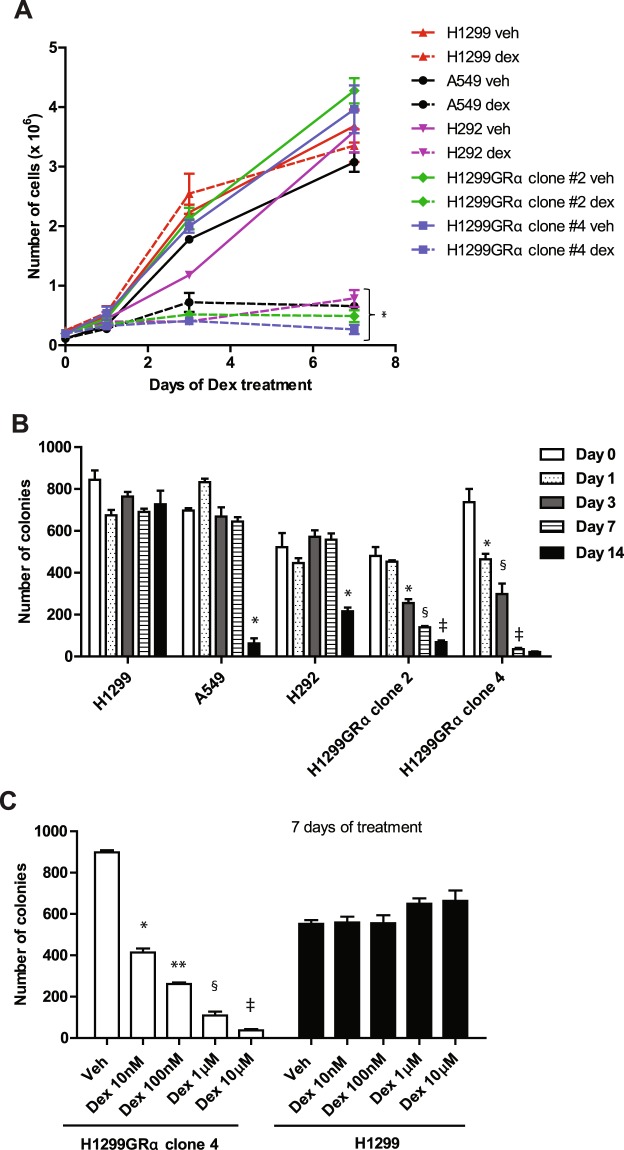
Effect of long-term Dex treatment on growth and colony forming ability of model lung adenocarcinoma cell lines. H292, A549, H1299, H1299GR Clone 2 and H1299GR Clone 4 cells were treated with either vehicle or Dex (100 nM) and cell growth was monitored by counting the viable cells using the Trypan blue dye exclusion assay (Panel A). The same cell lines were treated with Dex (100 nM) for the indicated days and then plated to measure colony formation without further treatment (Panel B). H1299GR Clone 4 cells or parental H1299 cells were treated with vehicle or the indicted concentrations of Dex for 7 days and plated to measure colony formation without further treatment (Panel C). Panel A: *P, 0.0001. Panel B (A549 cells): *P, 0.0001. Panel B (H292 cells): *P, 0.001. Panel B (H1299GRα clone2): *P, 0.001; ^**§**^P, 0.0005; ^‡^P, 0.015. Panel B (H1299GRα clone4): *P, 0.001; ^**§**^P, 0.0024; ^‡^P, 0.0001. Panel C: *P, 0.00004; **P, 0.0008; ^**§**^P, 0.0009; ^‡^P, 0.0005.

The highest plasma concentration of Dex when humans are administered a single dose of 4 mg of Dex is 100–200 nM^20^. Treatment with Dex (100 nM) for a duration of 7 days caused immediate and persistent growth arrest in all of the cell line models except for the parental H1299 cells, which expressed the lowest level of GR (Fig. 2A).

In the Dex-sensitive cell lines, the ability of Dex withdrawal to restore proliferation was measured by a colony formation assay performed immediately following different durations of Dex treatment, up to 14 days. Colony-forming ability was fully retained in the parental H1299 cells but it was lost progressively in the two GR-overexpressing recombinant H1299 cell lines. This effect was most pronounced in H1299GR Clone 4, which expressed the highest level of GR, with virtually complete loss of colony-forming ability by day 14 (Fig. 2B). In the A459 and H292 cell lines, which expressed moderate levels of GR, colony formation was considerably reduced between Day 7 and Day 14 of Dex treatment (Fig. 2B). The relationship between the dose of Dex used and colony-forming ability of GR overexpressing cells was examined using H1299GR Clone 4 following only 7 days of Dex treatment; the effect of Dex was dose-dependent, whereas even at the highest dose tested (10 µM) Dex had no effect on the parental H1299 cells (Fig. 2C).

**Figure 3:**
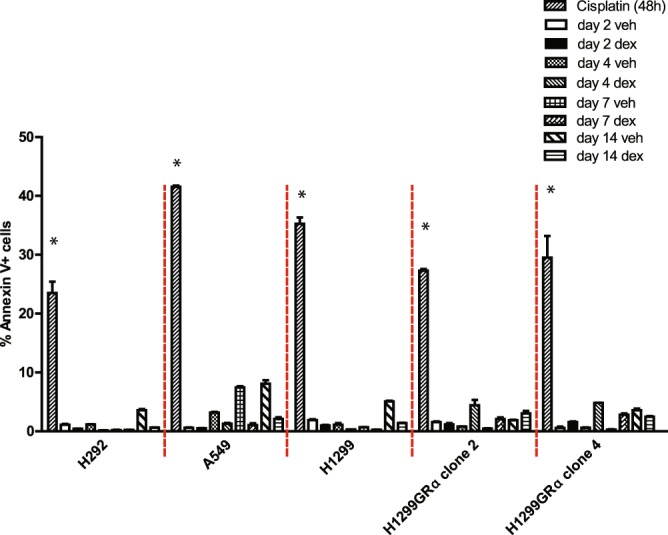
Lack induction of apoptosis in lung adenocarcinoma cells subjected to long-term Dex treatment. H292, A549, H1299, H1299 GR Clone 2 and H1299 GR Clone 4 cells were treated with either vehicle or Dex (100 nM) for the indicated number of days. Annexin V-positive cells were analyzed by flow cytometry. Cisplatin was used as the positive control. *P, 0.0001.

Extended treatment with Dex did not induce apoptosis in any of the cell line models as determined by staining for annexin V (Fig. 3).

### Dex induces senescence-associated lysosomal β-galactosidase activity, cell enlargement and loss of Ki67 expression in a GR status-dependent manner

**Figure 4:**
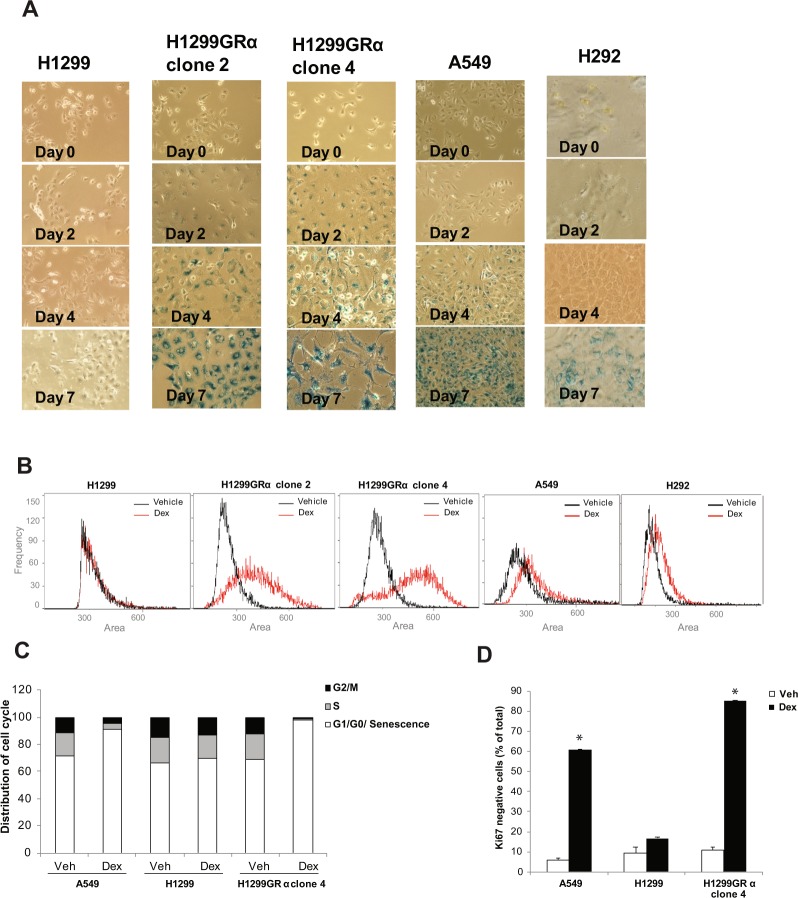
Induction of senescence phenotype in lung adenocarcinoma model cell lines by extended Dex treatment. H292, A549, H1299, H1299GR Clone 2 and H1299GR Clone 4 cells were treated with Dex (100 nM) for the indicated periods and the cells were stained to assess expression of β- galactosidase (blue staining) (Panel A). The same cell lines were treated with either vehicle or Dex (100 nM) for 4 days (H1299GR Clone 2 and H1299GR Clone 4) or for 7 days (H1299, H292, A549) and the distribution of relative cell size was determined by measuring the area of individual cells using the Amnis Mark II imaging cytometer (Panel B). A549, H1299 parental, and H1299GR clone 4 cells were plated at 20 percent confluence; the cells were then treated with either vehicle or Dex (100 nM) for 7 days, replenishing the treatments every 3 days. Cells were then analyzed by flow cytometry to measure cell cycle distribution (Panel C) and Ki67 negativity (Panel D). *P, 0.0004.

In sparsely plated recombinant H1299GR cell lines, Dex (100 nM) treatment resulted in positive staining for β-galactosidase activity, a hallmark of the senescence phenotype, beginning by Day 2 and increasing up to 7 days of treatment, in contrast to parental H1299 cells, which remained negative for β-galactosidase activity (Fig. 4A). In A549 and H292 cells, β-galactosidase activity was evident by Day 4 and became more intense by Day 7 (Fig. 4A).

Dex treatment also caused pronounced cell enlargement, also a hallmark of the senescence phenotype, in the two recombinant H1299 GR cell lines within 4 days of treatment (Fig. 4B). A similar effect was also evident in H292 and A549 cells after 7 days of treatment (Fig. 4B). In contrast, the parental H1299 cells did not show any change in size even after 7 days of treatment (Fig. 4B).

The senescence phenotype is known to be associated with suppression of S and mitotic phases of the cell cycle and Ki67 negativity. Therefore, we also examined by flow cytometery, the effect of 7-day treatment with Dex on cell cycle distribution (Fig. 4C) and Ki67 expression (Fig. 4D) in representative cell lines including A549 cells and the isogenic pair of parental H1299 (low GR) cells and H1299GR Clone 4 cells. Dex suppressed the S-phase and G2/M fractions in H1299GR Clone 4 cells and A549 cells and induced loss of Ki67 but did not appreciably affect either cell cycle distribution or Ki67 expression in the low GR parental H1299 cells.

### Dex causes transient induction of p21^Cip1^ and persistent induction of p27^Kip1^ but does not induce p16^INK4a^

**Figure 5:**
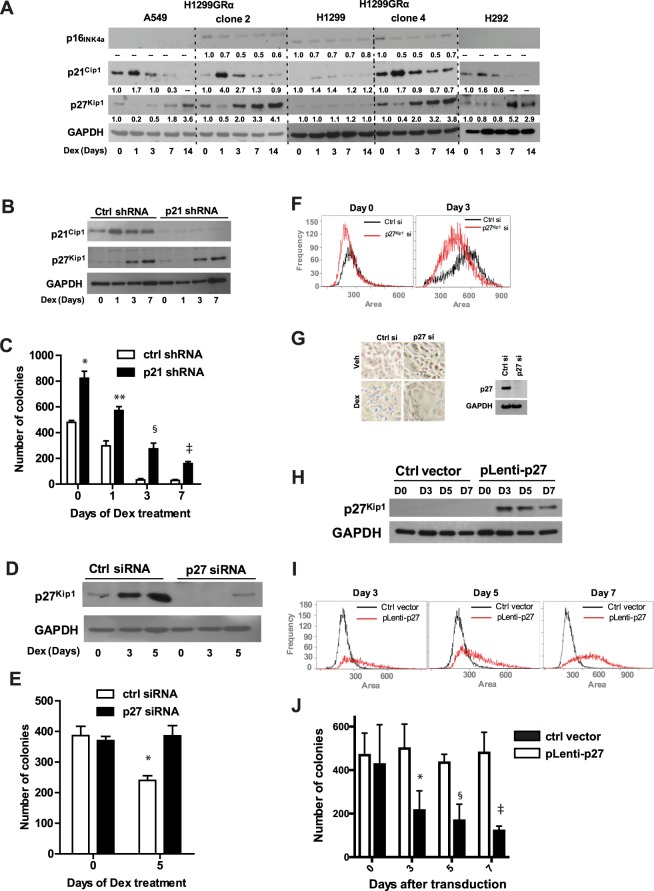
Expression and roles of p16^INK4a^, p21^Cip1^ and p27^Kip1^ in the response of lung adenocarcinoma cell line models to long-term treatment with Dex. H292, A549, H1299, H1299GR Clone 2 and H1299GR Clone 4 cells were treated with Dex (100 nM) for the indicated number of days and whole cell lysates were probed by western blot for p16^INK4a^, p21^Cip1^ and p27^Kip1^ with GAPDH as the loading control (Panel A). H1299GR Clone 4 cells were transduced with either non-targeted control shRNA or P21^Cip1^ shRNA and treated with Dex (100 nM) for the indicated number of days, when the cells were harvested and whole cell lysates were extracted and probed by western blot for p21^Cip1^, p27^Kip1^ and GAPDH (loading control) (Panel B). In parallel, cells treated as described for Panel B, were plated for colony formation in 6-well plates in triplicates without further treatment (Panel C). H1299GR Clone 4 cells were transfected with either non-targeted control siRNA or p27^Kip1^ siRNA and treated with Dex (100 nM) for the indicated number of days, when the cells were harvested and whole cell lysates were probed by western blot for p27^Kip1^ or GAPDH (loading control) (Panel D). In parallel, cells transfected as described for Panel D, and treated with Dex (100 nM) were plated on days 0 and 5 to measure colony formation in 6-well plates in triplicates without further treatment (Panel E) and also harvested on days 0 and 3 of treatment for measuring cell size (Panel F). H1299GR Clone 4 cells were transfected with either non-targeted control siRNA or p27^Kip1^ siRNA and 24 h later they were treated with Dex (100 nM) or vehicle for 3 days and the cells were stained to assess expression of β- galactosidase (blue staining) (Panel G, left); in parallel, the treated cells were lysed and analyzed by western blot for p27 expression (Panel G, right). H1299GR Clone 4 cells were transduced with either non-targeted control vector or pLenti-p27 expression vector and after the indicated number of days, whole cell lysates were probed by western blot for p27^Kip1^ or GAPDH (loading control) (Panel H). In parallel, cells were harvested for measuring the cell size (Panel I) and also plated to measure colony formation in 6-well plates in triplicate (Panel J). Panel C: *P, 0.0004; **P, 0.0006; ^§^P, 0.0008; ^‡^P, 0.00015. Panel E: *P, 0.016. Panel J: *P, 0.015; ^§^P, 0.00006; ^‡^P, 0.00007.

Next we undertook to examine whether any of the key proteins (p16^INK4a^, p21^Cip1^ and p27^Kip1^) representing the classical pathways of senescence might be induced by Dex in lung adenocarcinoma cells. Cell lysates were analyzed by western blots, following different durations of Dex (100 nM) treatment. There was no notable change in p16^INK4a^ in any of the cell line models (Fig. 5A). On the other hand, except in the parental H1299 cells, all of the cell line models showed strong induction of p21^Cip1^ within 24 h of Dex treatment, with the protein returning to near basal levels by Day 3 (Fig. 5A). In the recombinant H1299GR cell lines, p27^Kip1^ increased by Day 3 of treatment and continued to increase thereafter, remaining high on Day 14 (Fig. 5A). There was a relatively delayed increase in p27^Kip1^ in A549 and H292 cells (Fig. 5A). However, there was no change in p27^Kip1^ expression in parental H1299 cells.

### Depletion of p21^Cip1^ does not affect the ability of Dex to induce p27^Kip1^ or loss of proliferation potential

To test whether the transient induction of p21^Cip1^ by Dex was linked to the effect of Dex on proliferation potential or p27^Kip1^ induction, lentiviral shRNA was used to deplete p21^Cip1^ in H1299 Clone 4 cells (Fig. 5B). Dex induced p27^Kip1^ regardless of expression or induction of p21^Cip1^ (Fig. 5B). Moreover, whereas depletion of p21^Cip1^ did increase the basal level of colony formation, it did not affect the ability of Dex treatment to progressively suppress colony forming ability following Dex withdrawal (Fig. 5C).

### Induction of senescence phenotype by Dex in GR-overexpressing cells is mediated by p27^Kip1^

Depletion of p27^Kip1^ using transient transfection of siRNA in GR overexpressing H1299 Clone 4 cells (western blot, Fig. 5D) rescued colony formation in Dex-treated cells (Fig. 5E). Further, the average cell size was smaller in p27^Kip1^ depleted cells compared to cells transfected with control siRNA (Fig. 5F). Depletion of p27^Kip1^ also blocked the ability of Dex to induce β-galactosidase activity (Fig. 5G). Finally, in the same cells, in the absence of Dex, ectopic overexpression of p27^Kip1^ (Fig. 5H) caused cell expansion (Fig. 5I) and also suppressed colony formation (Fig. 5J). These complementary loss- and gain-of-function experiments establish that accumulation of p27 ^Kip1^ in response to chronic Dex treatment is necessary and sufficient for the effect of Dex on the ability of the cells to proliferate as well as the ability Dex to induce a senescence like phenotype.

### Induction of p27^Kip1^ by Dex is independent of its ability to regulate FOXO3 and FOXO4

**Figure 6:**
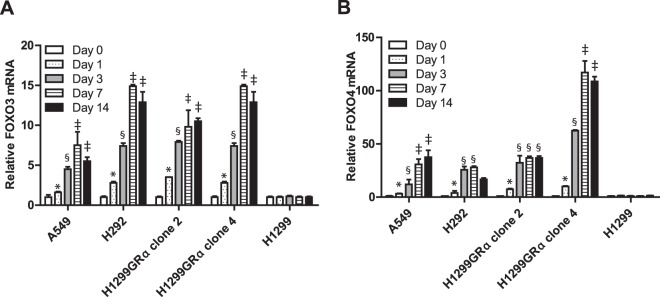
Induction of mRNAS for FOXO3 and FOXO4 by Dex in lung adenocarcinoma cells. H292, A549, H1299, H1299GR Clone 2 and H1299GR Clone 4 were treated with Dex (100 nM) for the indicated number of days, RNA was extracted and mRNA expression levels of FOXO3 (Panel A) and FOXO4 (Panel B) were measured using real time RT-PCR. Panel A (A549): *P, 0.027; ^**§**^P, 0.009; ^‡^P, 0.04. Panel A (H292): *P, 0.003; ^**§**^P, 0.0001; ^‡^P, 0.0001. Panel A (H1299GRα clone2): *P, 0.0001; ^**§**^P, 0.0001; ^‡^P, 0.05. Panel A (H1299GRα clone4): *P, 0.0001; ^**§**^P, 0.0001; ^‡^P, 0.0001. Panel B (A549): *P, 0.05; ^**§**^P, 0.05; ^‡^P, 0.002. Panel B (H292): *P, 0.03; ^**§**^P, 0.0001. Panel B (H1299GRα clone2): *P, 0.01; ^**§**^P, 0.0001. Panel B (H1299GRα clone4): *P, 0.012; ^**§**^P, 0.0001; ^‡^P, 0.0001.

**Figure 7:**
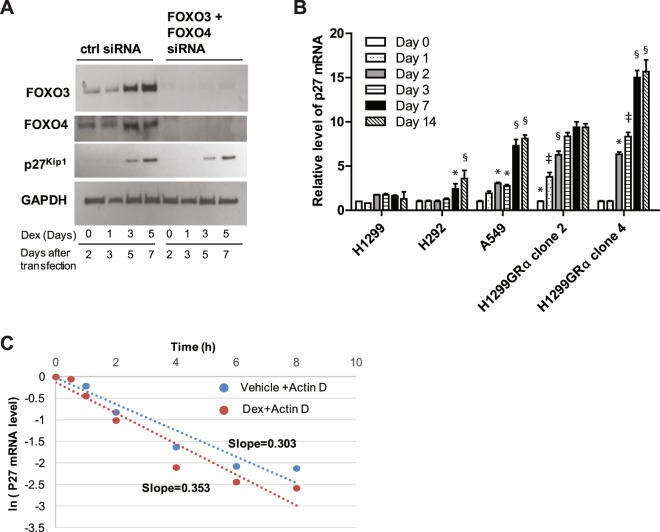
Mechanism of accumulation of p27^Kip1^ in response to long-term Dex treatment in high GR expressing cells. H1299GR Clone 4 cells were transfected with either non-targeted control siRNA or a combination of FOXO3 and FOXO4 siRNAs and treated with Dex (100 nM) for the indicated number of days, when whole cell lysates were probed by western blot for FOXO3, FOXO4, p27^Kip1^ and GAPDH (loading control) (Panel A). H292, A549, H1299, H1299GR Clone 2 and H1299GR Clone 4 cells were treated with Dex (100 nM) for the indicated number of days, when RNA was extracted and relative expression levels of p27^Kip1^ mRNA were measured using real time RT-PCR (Panel B). H1299GR Clone 4 cells were pre-treated with vehicle or Dex (100 nM) for 72 h. The cells were then treated with 1ug/ml of actinomycin D and at the time points indicated, RNA was harvested from the cells and p27^Kip1^ mRNA was measured by real time RT-PCR to determine p27^Kip1^ mRNA turnover rates. The mRNA values are plotted relative to the values at the time of addition of actinomycin D (Panel C). Panel B (H292): *P, 0.01; ^**§**^P, 0.008. Panel B (A549): *P, 0.02; ^**§**^P, 0.0001. Panel B (H1299GRα clone2): *P, 0.0001; ^‡^P, 0.0007, ^**§**^P, 0.001. Panel B (H1299GRα clone4): *P, 0.0001; ^‡^P, 0.001; ^**§**^P, 0.0001.

FOXO3 and FOXO4 are known to be induced by glucocorticoids in some cell types^21^ and in other cell types, a FOXO3/FOXO4 axis is also known to regulate the p27^Kip1^ gene^22,23^. Dex did transcriptionally activate both FOXO3 and FOXO4 in all of the lung adenocarcinoma cell lines in this study except for the parental (low GR) H1299 cells; the mRNAs for FOXO3 and FOXO4 in all of the sensitive cells increased within 24 h of Dex treatment and reached plateau levels by 3 to 7 days of treatment (Fig. 6A and B). This was reflected by induction of FOXO3 and FOXO4 proteins by Dex (tested in H1299GR Clone 4 cells in Fig. 7A). However, combined depletion of FOXO3 and FOXO4 using targeted siRNA failed to influence the ability of Dex to induce p27^Kip1^ (Fig. 7A). These results exclude a role for a FOXO3/FOXO4 axis in regulation of p27^Kip1^ in lung adenocarcinoma cells.

### Dex induces progressive accumulation of p27^Kip1^ mRNA in GR-overexpressing cells without affecting mRNA stability

In all of the Dex-sensitive lung adenocarcinoma models used in this study, Dex induced a progressive accumulation of p27^Kip1^ mRNA, in contrast to the parental H1299 (low GR) cells (Fig. 7B). The rate of accumulation of p27^Kip1^ mRNA upon Dex treatment was relatively slow, with ≤2-fold increase in 24 h.

The stability of p27 mRNA was measured in H1299GR Clone 4 cells after blocking *de novo* mRNA synthesis using actinomycin D (Fig. 7C). Treatment with Dex did not affect the half-life of p27^Kip1^ mRNA which was about 2 h (Fig. 7C). This result indicates that the Dex-induced accumulation of p27^Kip1^ mRNA noted above is the result of increased transcription of the p27^Kip1^ gene.

### Dex suppresses growth of GR-overexpressing cell line xenografts in SCID mice together with induction of p27^Kip1^

To examine the effect of Dex on GR-overexpressing lung adenocarcinoma tumors *in vivo*, we first used isogenic tumor xenografts derived from parental H1299 (low GR) cells and H1299GR Clone 4 (high GR) cells. As in contrast to humans, Dex is rapidly cleared in mice, it was necessary to implant slow release Dex pellets in the mice to maintain a constant level of Dex in the circulation. Notably, in contrast to humans, mice on extended Dex treatment are prone to paralytic ileus as major adverse side effect; this limited the Dex dose to 2.5 mg pellets and the duration of the experiment after implantation of Dex pellets to a little over 2 weeks. The median plasma Dex level in mice, measured one week after pellet implantation, was 181.5 nM, comparable to pharmacological plasma Dex levels in humans^20^.

**Figure 8:**
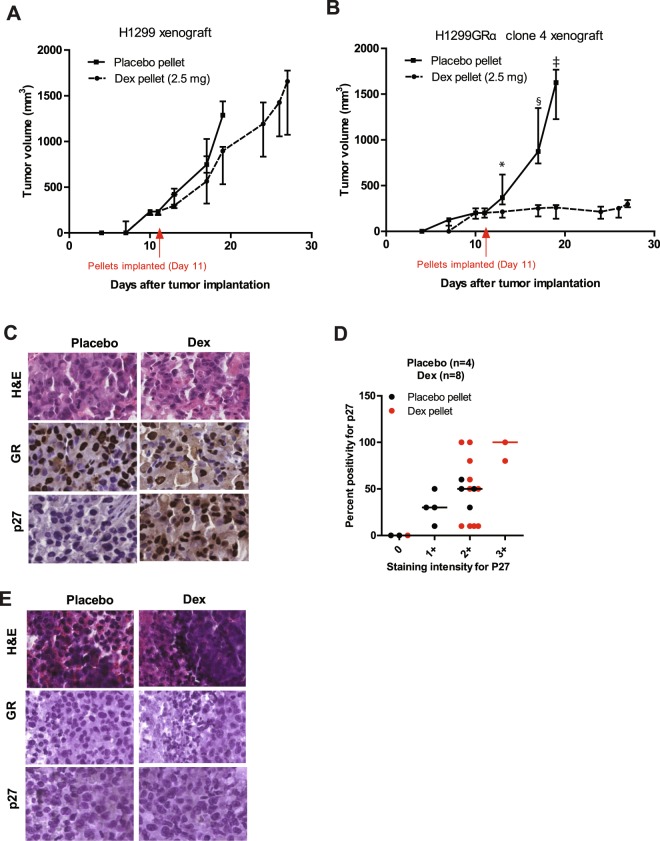
Effect of Dex on mouse tumor xenografts expressing low or high levels of GR. SCID mice bearing xenografts of parental H1299 cells (Panel A) or H1299GR Clone 4 cells (Panel B) were implanted with either placebo or Dex (2.5 mg) slow-release pellets at the time indicated by the arrow. The tumor growth was monitored via caliper measurements and tumor volumes were calculated using the formula noted in the Methods section. At the time of sacrifice, residual H1299GR Clone 4 tumors were harvested from the placebo (Panel C, left) and Dex (Panel C, right) treated mice and stained to ensure GR expression and to quantify p27^Kip1^ by immunohistochemistry (Panel D). The scoring criteria based on staining intensity and percent positivity, as outlined in the Methods section. The parental H1299 cell tumor xenografts harvested at the end of placebo or Dex treatments in Panel A were also examined by immunohistochemistry for GR and p27^Kip1^ expression (Panel E). Panel B: *P, 0.08; ^**§**^P, 0.016; ^‡^P, 0.0015.

The Dex pellets (or placebo control pellets) were implanted after the tumor xenografts had taken. The growth of the H1299 tumors was comparable in the presence of Dex vs placebo pellets (Fig. 8A). In contrast, although the H1299GR Clone 4 tumors grew as well as the H1299 tumors in placebo treated mice, Dex treatment virtually completely suppressed tumor growth (Fig. 8B). Residual tumors from all of the mice harboring the H1299GR Clone 4 tumors were harvested at the time of sacrifice and were examined by immunohistochemistry. The tumors maintained GR expression but only showed elevated p27^Kip1^ expression in response to Dex treatment, in contrast to placebo (Fig. 8C). In the residual H1299GR Clone 4 tumors, Dex treatment was strongly associated with elevation of p27^Kip1^, both in staining intensity and percent positivity (Fig. 8D). As an additional control, the tumor xenografts generated from the parental H1299 cells consistently showed low or absent expression of both GR and p27^Kip1^ at termination of treatments in Fig. 8A, both in the placebo and Dex treated groups as seen in the representative immunohistochemistry images shown (Fig. 8E). There was not an appreciable change in the average mouse weights in any of the treatment groups (Supplementary Tables 1 and 2).

## Discussion

The effect of long-term treatment with glucocorticoids on lung adenocarcinoma cells has not been previously reported. Our studies demonstrate that Dex induces a progressive loss of proliferation potential of lung adenocarcinoma cells that is associated with a senescence phenotype, characterized by β-galactosidase activity, cell enlargement and Ki67 negativity. This effect was observed in cell line models of variable p53 or KRAS status and its occurrence and time of onset was entirely dependent on the GR expression status, Dex dose and duration of exposure to Dex. The effect of Dex, observed using isogenic GR-high and GR-low cells, was most pronounced at GR expression levels corresponding to the top 30 percent in clinical tumors as measured by accurate quantification of GR mRNA, which in model cells corresponded to relative GR protein expression. Tissue microarray data showed a broad range of distribution of GR expression in lung adenocarcinoma tumors with about half of all tumor sections showing at least a portion of the malignant cells with relatively high GR expression.

In malignant cells, irreversible cell-cycle blockade that is induced in response to stress stimuli or the activity of tumor suppressors and oncogenes, is mediated by the INK4 family and Cip/Kip family of CDK inhibitors^24,25^. However, the long-term Dex-induced loss of proliferation potential in lung adenocarcinoma cells observed in this study did not occur through canonical senescence pathways involving these proteins such as the p16^INK4a^/Rb pathway, the p19^ARF^/p53/p21^Cip1^ pathway and the PTEN/p27^Kip1^ pathway, despite the transient early induction of p21^Cip1^ or the functional linkage between the phenotypic effects of Dex and accumulation of p27^Kip1^. Moreover, p27^Kip1^ induction by Dex did not entail regulatory mechanisms of p27^Kip1^ observed in other cell types including transcriptional activation by FOXO3/FOXO4^22,23^. Dex induced a progressive accumulation of p27^Kip1^ mRNA that was not associated with a change in mRNA stability. Our studies indicate that in lung adenocarcinoma cells, a relatively slow rate of transcriptional activation of the p27^Kip1^ gene that occurs at relatively high GR levels accounts for the progressive accumulation of p27^Kip1^ in response to Dex treatment. Loss- and gain-of-function experiments clearly established that it was the accumulation of p27^Kip1^ induced by chronic exposure to Dex that caused progressive loss of the ability of the cells to proliferate and excluded p21^Cip1^ in this role. The cumulative level of p27^Kip1^ induced by Dex as a function of GR level, Dex dose and treatment duration is likely the key factor in causing permanent cell cycle blockade because p27^Kip1^ can only effectively inhibit its target CDK2/E-A kinases when expressed at a level above that at which p27^Kip1^ is sequestered in a stabilizing complex with CDK4-6/D^26^. The upstream mechanism of regulation of p27^Kip1^ by Dex, while not conforming to known mechanisms of regulation p27^Kip1^, is consistent with a weak direct gene activation by GR in response to Dex. As steroid receptors typically regulate their target genes from distant binding sites in the chromatin (10–50 kb), future studies should be directed at elucidating the precise mechanism of p27^Kip1^ gene activation by Dex.

Most recent observations^10^ of the survival benefit of Dex treatment given peri-operatively to 588 patients with non-small-cell lung cancer with a 5-year postoperative follow-up as well as the currently open prospective clinical trial to validate this effect, is significant in the context of our findings. The mechanism for this survival benefit has not been established and it has been speculated that the effect may be due to relief of postoperative stress, which may inhibit the cytotoxic effects of natural killer cells and the activity of T cells, leading to immunosuppression. However, this would only be a brief period of stress and there was no indication of such Dex-associated benefits following ovarian cancer and colon cancer surgery^27,28^. More important, the direct effect of Dex on the tumor cells was not investigated and there was no mechanistic rationale to stratify patients for Dex treatment. In light of our findings that the higher the tumor cell GR expression, the earlier is the onset of permanent cell cycle blockade, it is possible that the duration of perioperative Dex treatment was adequate, though not optimal, to produce a significant survival benefit by direct action on the tumors. It is therefore also noteworthy that the selective tumor p27^Kip1^ induction and tumor growth inhibition associated with Dex treatment of mice bearing the isogenic GR-high tumor xenografts vs. GR-low tumors, demonstrates that systemic factors do not play a significant role in the antitumor effects of Dex and that tumor GR status is the principal determinant of tumor sensitivity to Dex. The combined mechanistic and pre-clinical data also demonstrate that p27^Kip1^ may be used clinically as a biomarker for the induction of permanent cell-cycle blockade in lung adenocarcinomas following long-term treatment with Dex.

Based on our studies, incorporating extended treatment with a moderate dose of Dex, into the standard of care pemetrexed chemotherapy regimen, may offer an innocuous but significantly beneficial treatment option for a large number of lung cancer patients stratified on the basis of tumor GR status. High GR-expressing tumor cell populations are predictably protected against pemetrexed-based chemotherapy due to reversible G1 arrest by Dex, which is part of the standard chemotherapy regimen^7^ but those cell populations should eventually exhibit permanent cell cycle blockade upon extending Dex treatment. It has also been proposed that induction of cellular senescence by therapeutic agents may contribute to tumor sensitivity to the treatments, by mobilizing immune response against the tumors^29^. The rationale for current attempts to combine standard of care chemotherapy and immunotherapy in treating lung adenocarcinoma capitalizes on this concept of enhanced recognition of the senescent tumor cells by the immune system^30^. Although our studies would predict that only tumors uniformly expressing relatively high levels of GR would respond optimally to the anti-proliferative effect of long-term treatment with Dex, the senescence phenotype induced by Dex in tumors that are heterogeneous in GR expression should also elicit an anti-tumor immune response following Dex withdrawal. As Dex may cause some reversible immunosuppression even at moderate doses, when administered for longer periods, a reasonable combination treatment to investigate would be to administer extended Dex treatment prior to immune activating therapies. Immediate translational clinical trials of our pre-clinical findings (underway at our institution) pose few logistical challenges as the treatment is safe, the patients may be stratified by prior examination of tumor GR expression in multiple core biopsies, routine radiological monitoring could be used to examine the effect of Dex and p27^Kip1^ could be measured as a biomarker of Dex-induced senescence.

## Electronic supplementary material

Dataset 1

Original Autorads

## Data Availability

All data generated or analyzed during this study are included in this published article.
