# Supplementary material for: Chronic p27Kip1 Induction by Dexamethasone Causes Senescence Phenotype and Permanent Cell Cycle Blockade in Lung Adenocarcinoma Cells Over-expressing Glucocorticoid Receptor
Source: Sci Rep. 2018 Oct 30;8:16006. doi: 10.1038/s41598-018-34475-8 (PMC6207728; doi:10.1038/s41598-018-34475-8)
Supplement: Supplementary file 1 — Dataset 1 [file 41598_2018_34475_MOESM1_ESM.pdf]

Supplementary Tables

Manuscript title: Chronic $p27^{Kip1}$ Induction by Dexamethasone Causes Senescence Phenotype and Permanent Cell Cycle Blockade in Lung Adenocarcinoma Cells Over-expressing Glucocorticoid Receptor

Authors: Mugdha Patki, Thomas McFall, Rayna Rosati, Yanfang Huang, Agnes Malysa, Lisa Polin, Abigail Fielder, Mike R. Wilson, Fulvio Lonardo, Jessica Back, Jing Li, Larry H. Matherly, Gerold Bepler and Manohar Ratnam

Supplementary Table 1: Monitoring body weights of SCID mice harboring H1299 tumor xenografts in Figure 8A

Days After Tumor Implantation	0	12	13	14	15	16	17	18	19	20	21	22	23	24	25	26	27
Placebo Percent body weight lost/gained	0	-4.4	-4	-2	-2	-4	0.4	0.4	9.15	8.75	-	-	-	-	-	-	-
Dex Percent body weight lost/gained	0	-4.3	-3.7	2.4	2.4	-3.7	4.75	4.75	4.75	0	-1.3	4.75	-3.2	-3.7	2.9	-3.7	-1.3

Supplementary Table 2: Monitoring body weights of SCID mice harboring H1299GRα tumor xenografts in Figure 8B

Days After Tumor Implantation	0	12	13	14	15	16	17	18	19	20	21	22	23	24	25	26	27
Placebo Percent body weight lost/gained	0	0	-1.7	0	-6.05	-1.7	-1.7	-1.7	0	0	-	-	-	-	-	-	-
Dex Percent body weight lost/gained	0	-3.9	-3.9	+2	0	-3.9	0	-3.9	-7.85	-11.7	-9.75	-11.7	-13.7	-7.75	-3.75	-7.75	-11.7
