# Supplementary material for: Chronic p27Kip1 Induction by Dexamethasone Causes Senescence Phenotype and Permanent Cell Cycle Blockade in Lung Adenocarcinoma Cells Over-expressing Glucocorticoid Receptor
Source: Sci Rep. 2018 Oct 30;8:16006. doi: 10.1038/s41598-018-34475-8 (PMC6207728; doi:10.1038/s41598-018-34475-8)

Manuscript title:

Chronic p27[Kip1] Induction by Dexamethasone Causes Senescence Phenotype and Permanent Cell Cycle Blockade in Lung Adenocarcinoma Cells Over-expressing Glucocorticoid Receptor

Authors:

Mugdha Patki, Thomas McFall, Rayna Rosati, Yanfang Huang, Agnes Malysa, Lisa Polin, Abigail Fielder, Mike R. Wilson, Fulvio Lonardo, Jessica Back, Jing Li, Larry H. Matherly, Gerold Bepler and Manohar Ratnam

Scans of original autorads for the cropped western blot data in the manuscript Figures

Please Note: When a western blot is probed for GAPDH loading control (example, Figure 1B), we typically use a single film to obtain multiple exposures by sliding the blot to different positions on the same film. This helps to avoid overexposure of the relatively strong GAPDH bands.

Each cropped image in the manuscript is from a single western blot, without any splicing of sample lanes.

Figure 1B_GR

GR

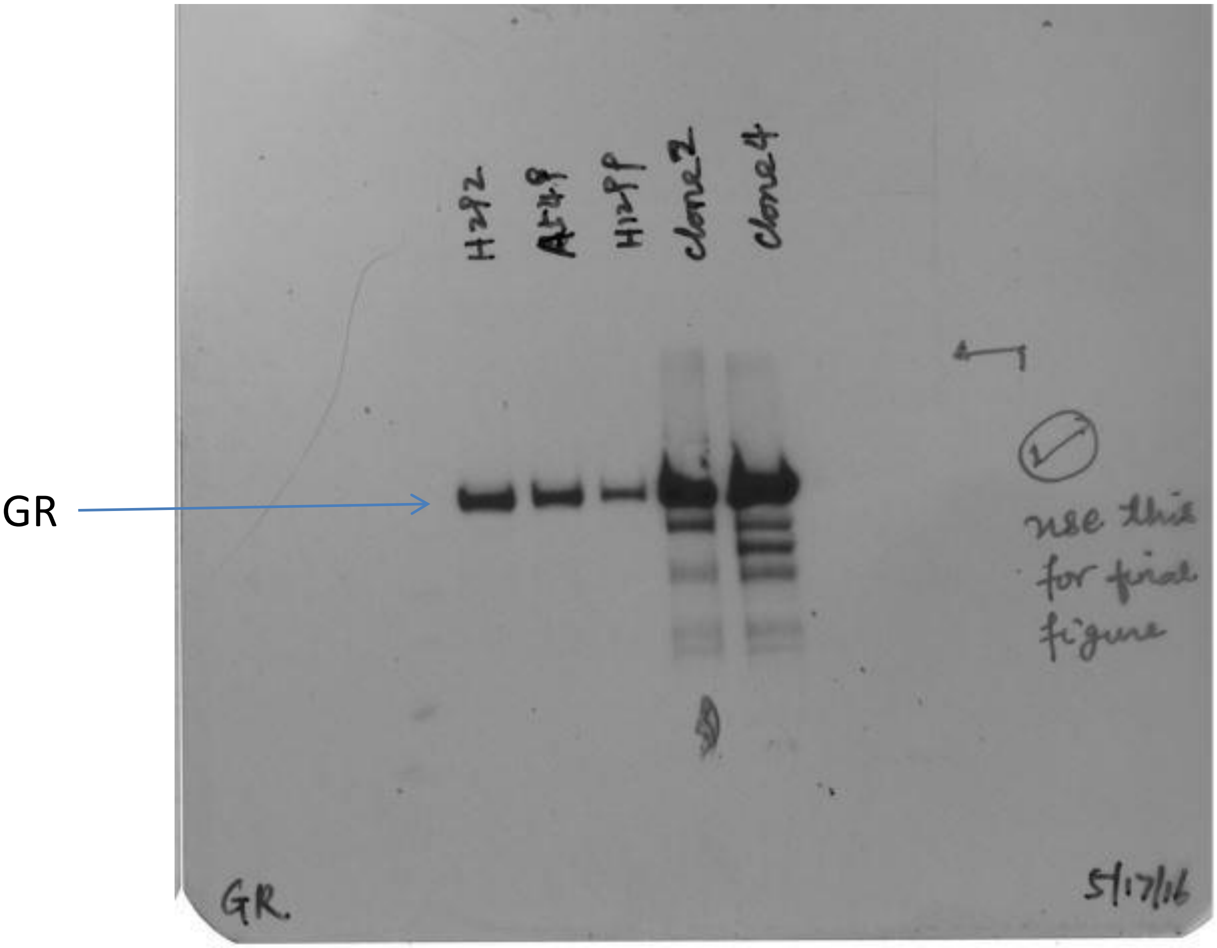

Figure 1B_GAPDH

GAPDH

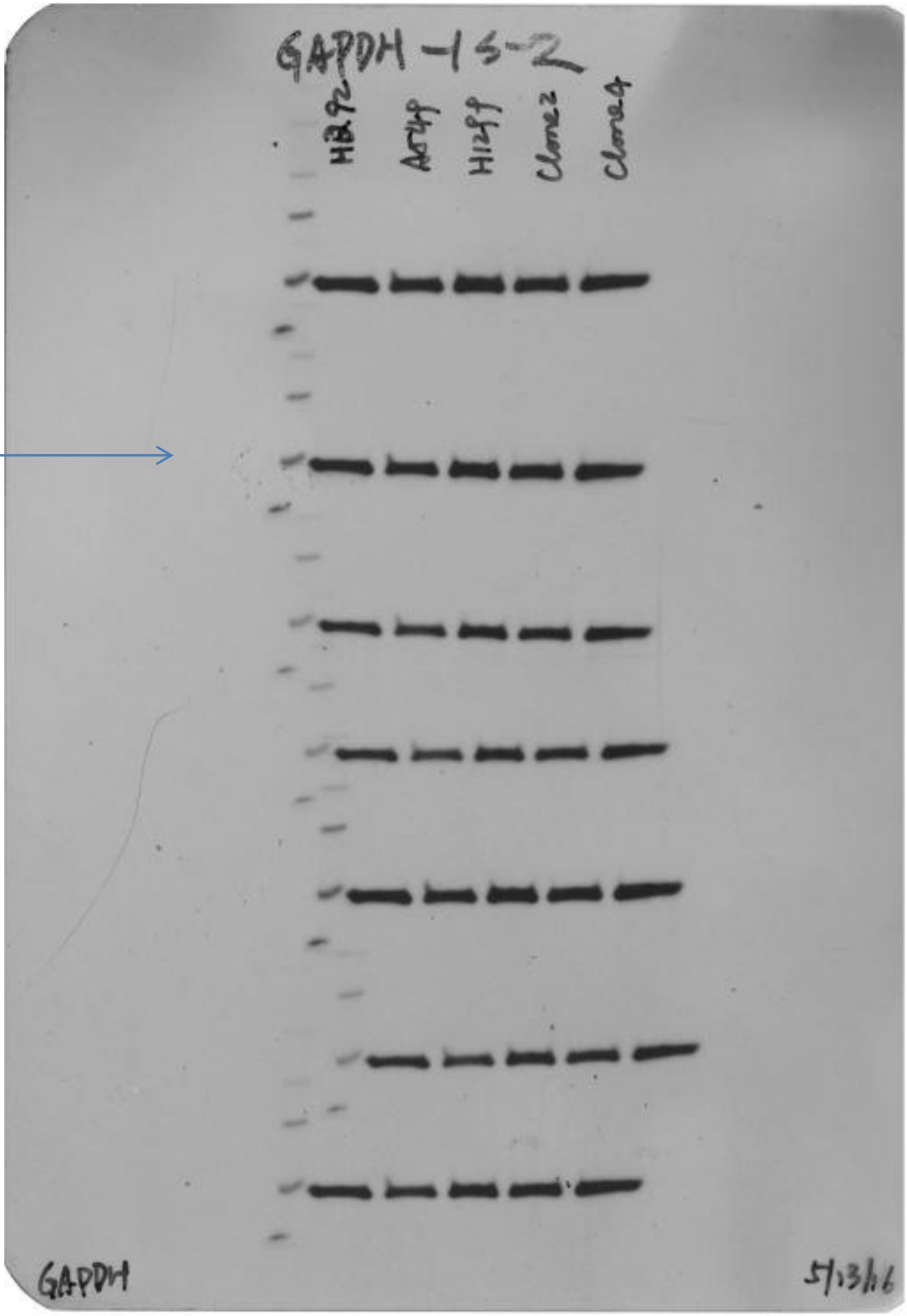

Figure 5A_A549_Clone2_H1299_Clone4_p16

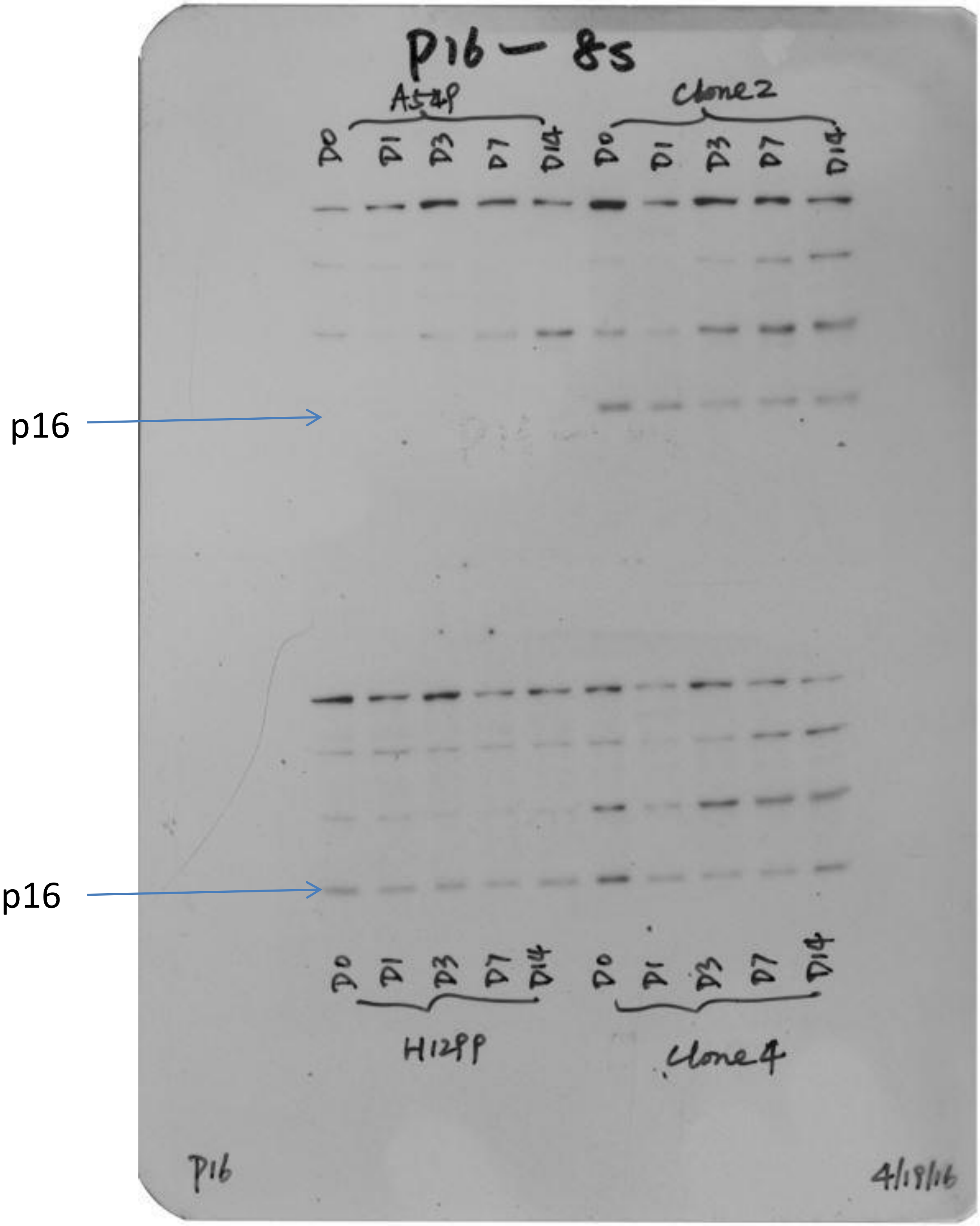

p16

p16

Figure 5A_A549_Clone2_H1299_Clone4_p21

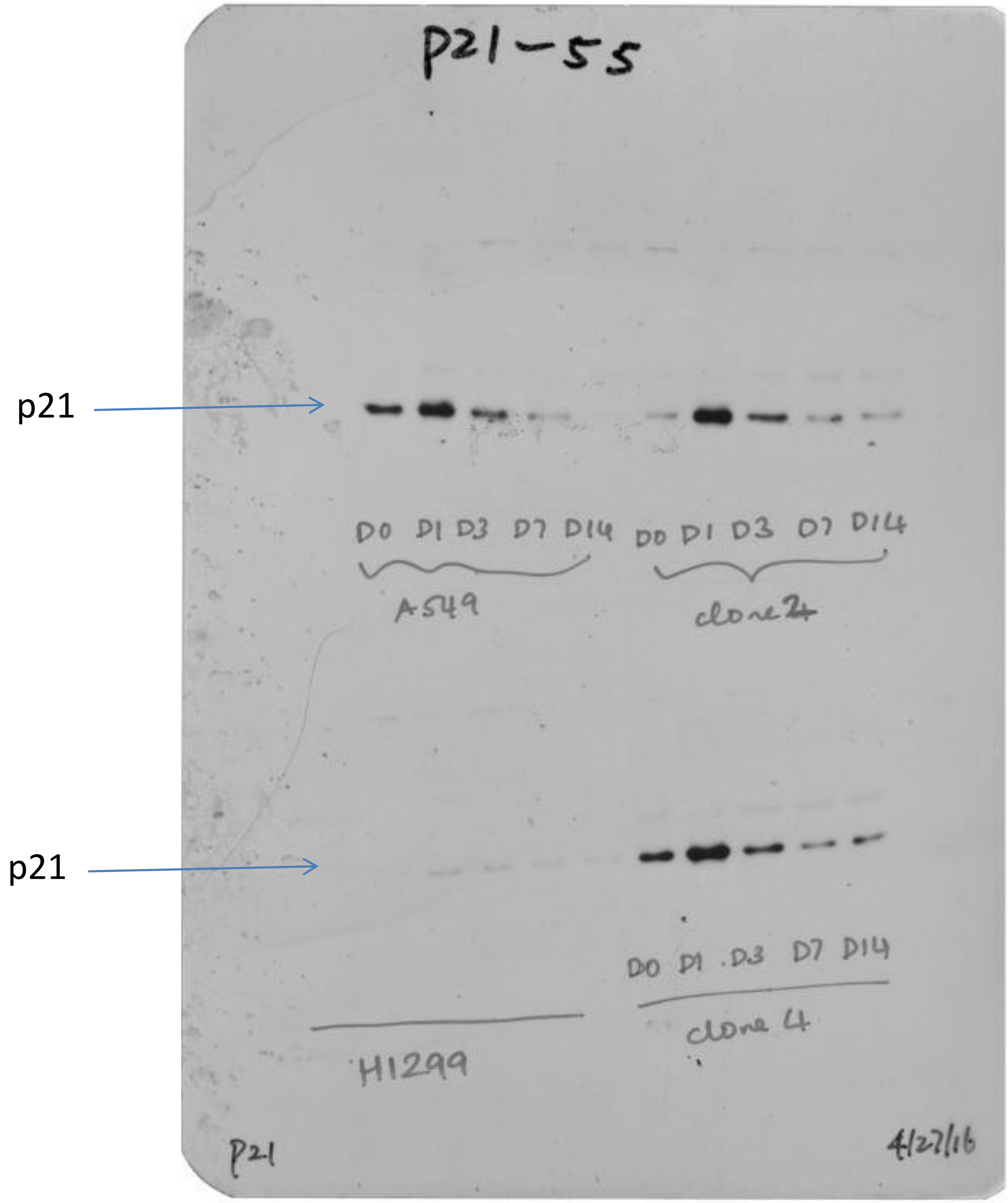

Figure 5A_A549_Clone2_H1299_Clone4_p27

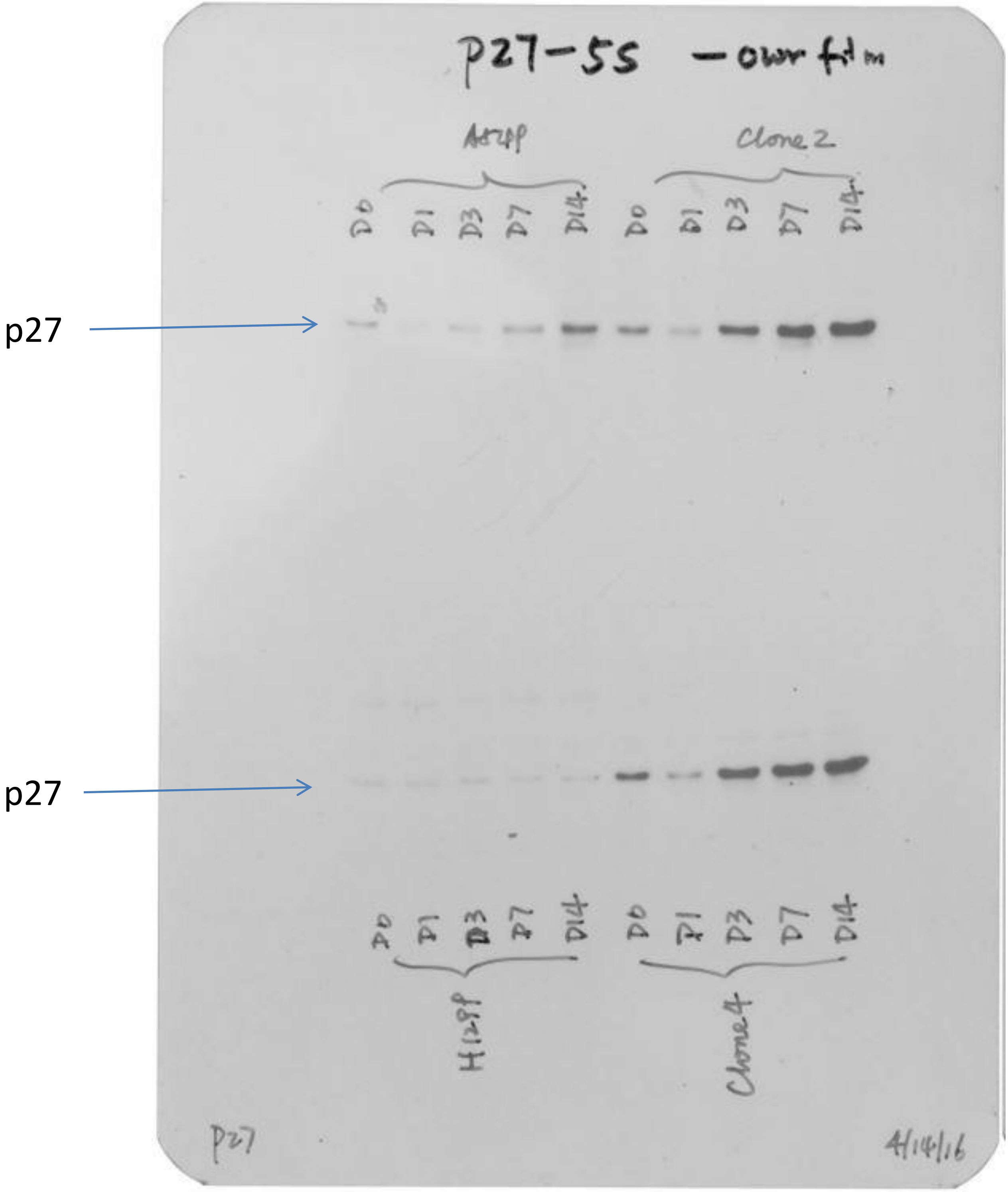

Figure 5A_A549_Clone2_GAPDH

GAPDH

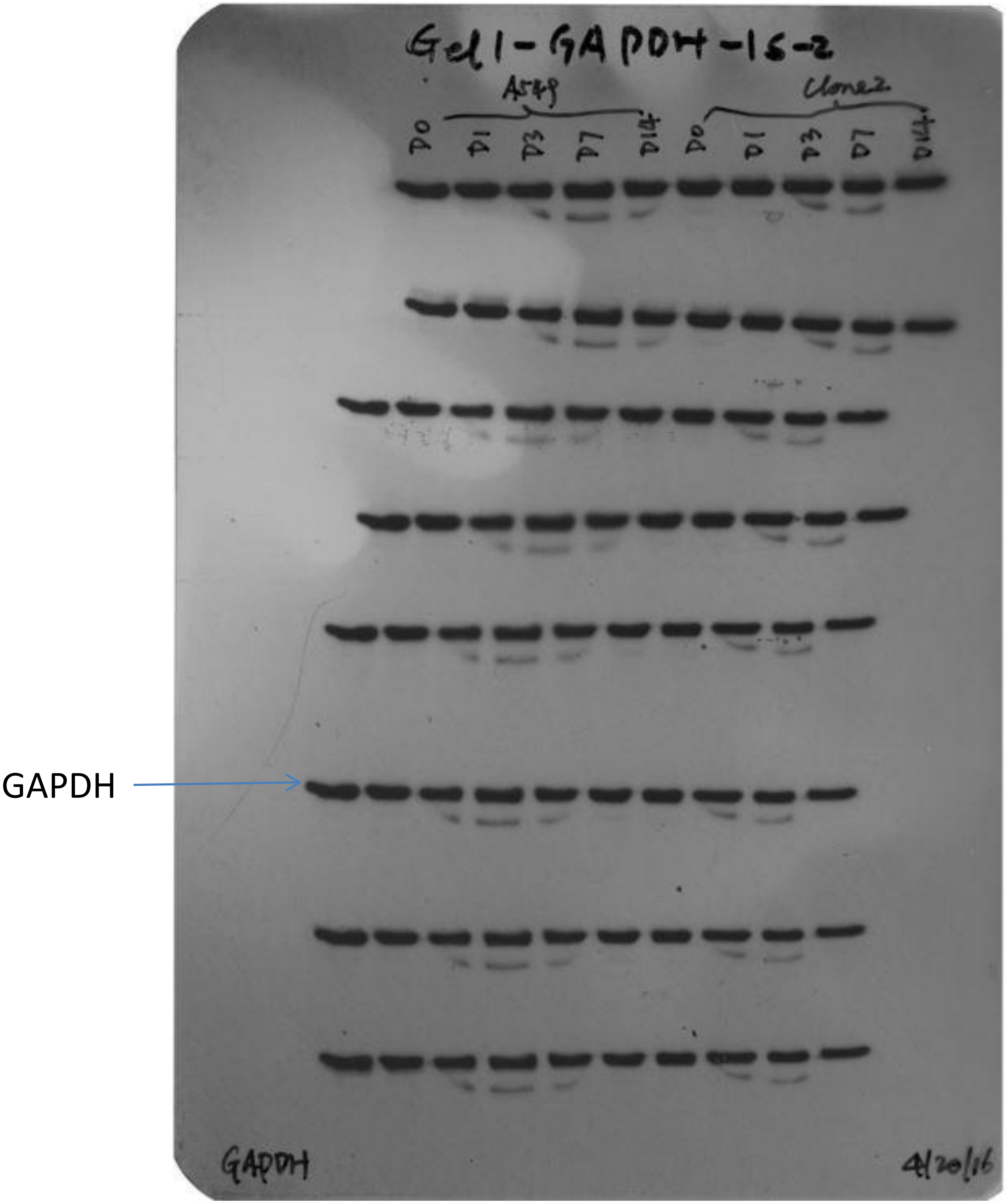

Figure 5A_H1299_Clone4_GAPDH

GAPDH

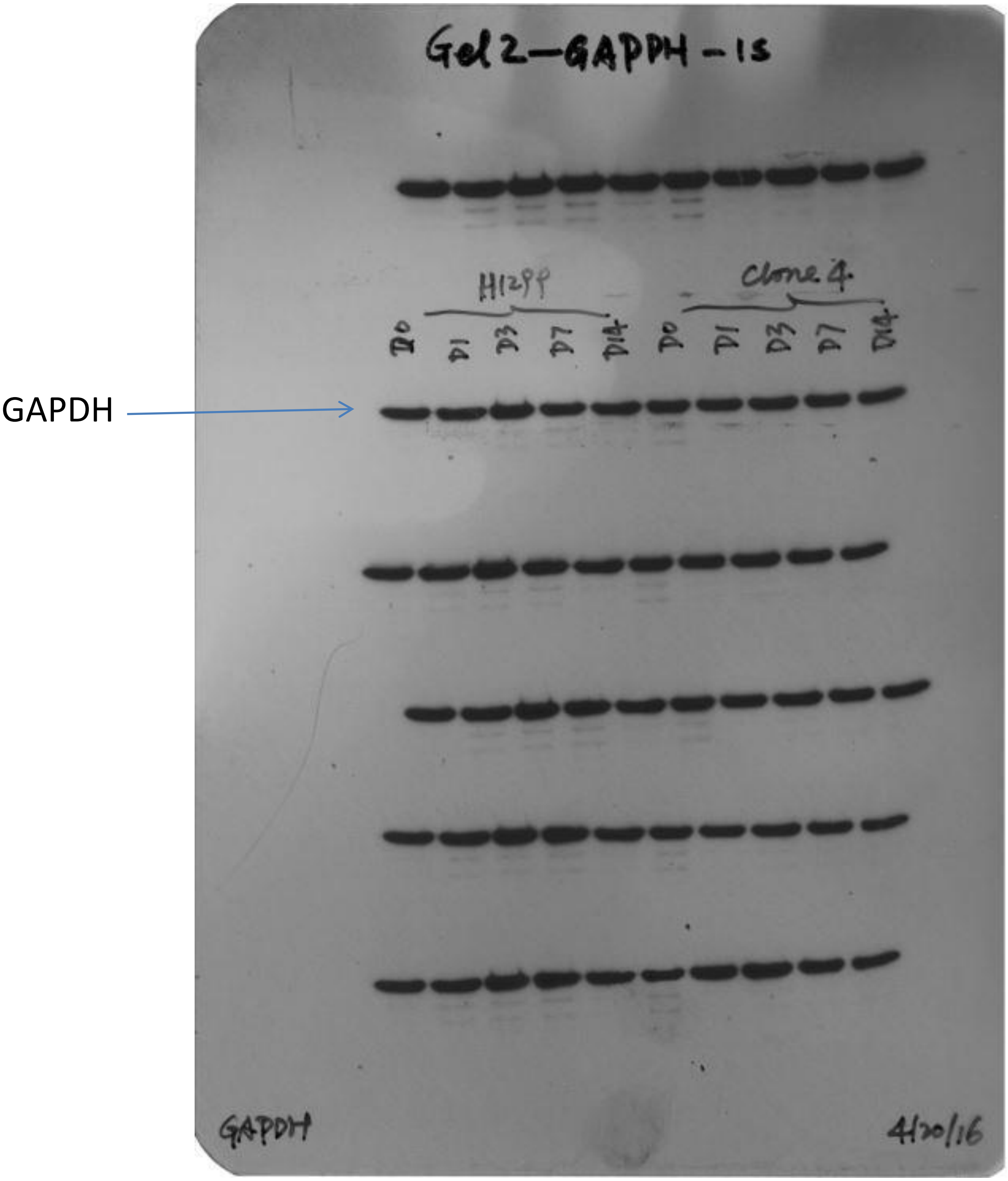

Figure 5A_H292_p21_p16

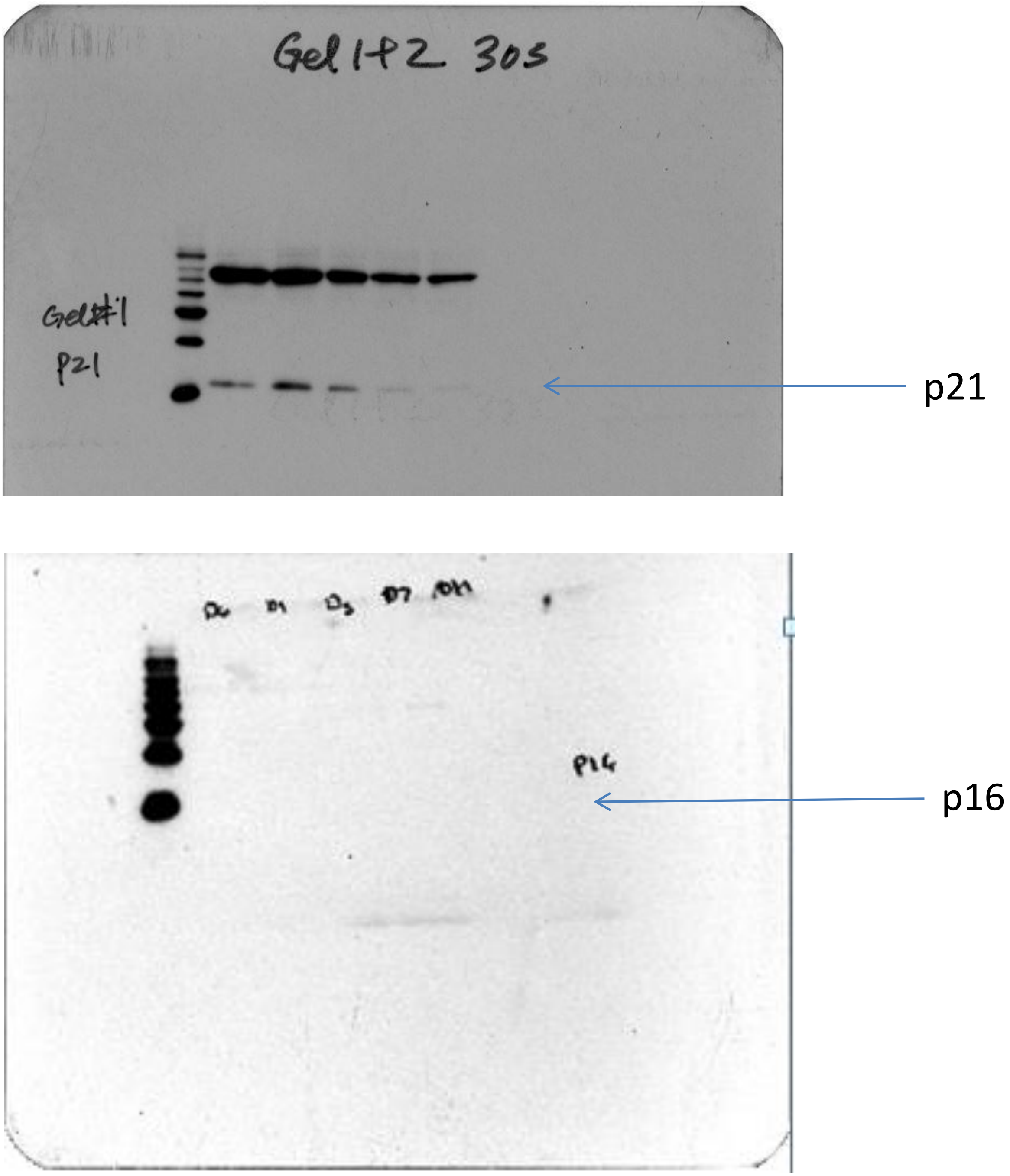

Figure 5A_H292_p27_GAPDH

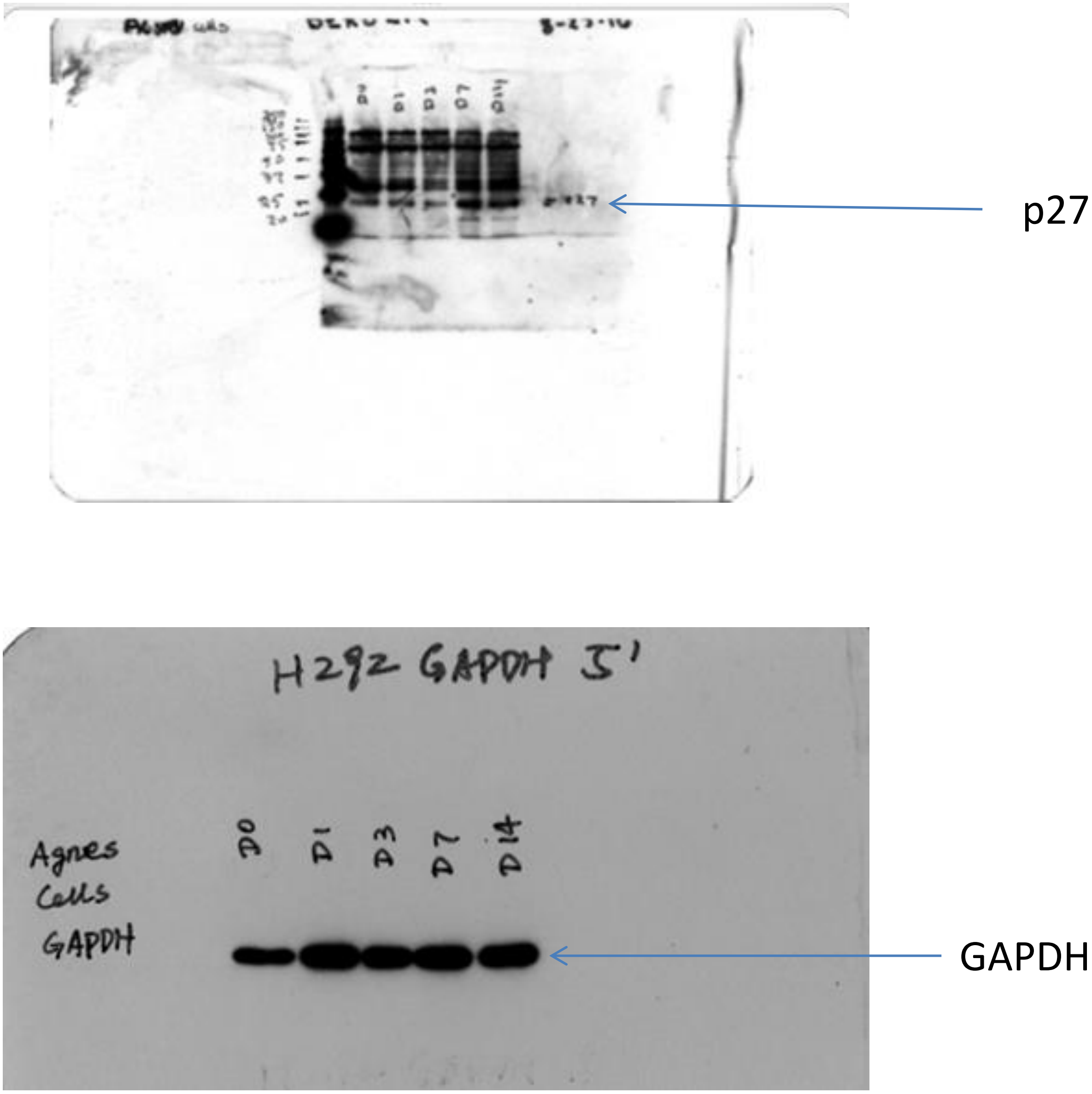

Figure 5B_Clone4_p21+p27

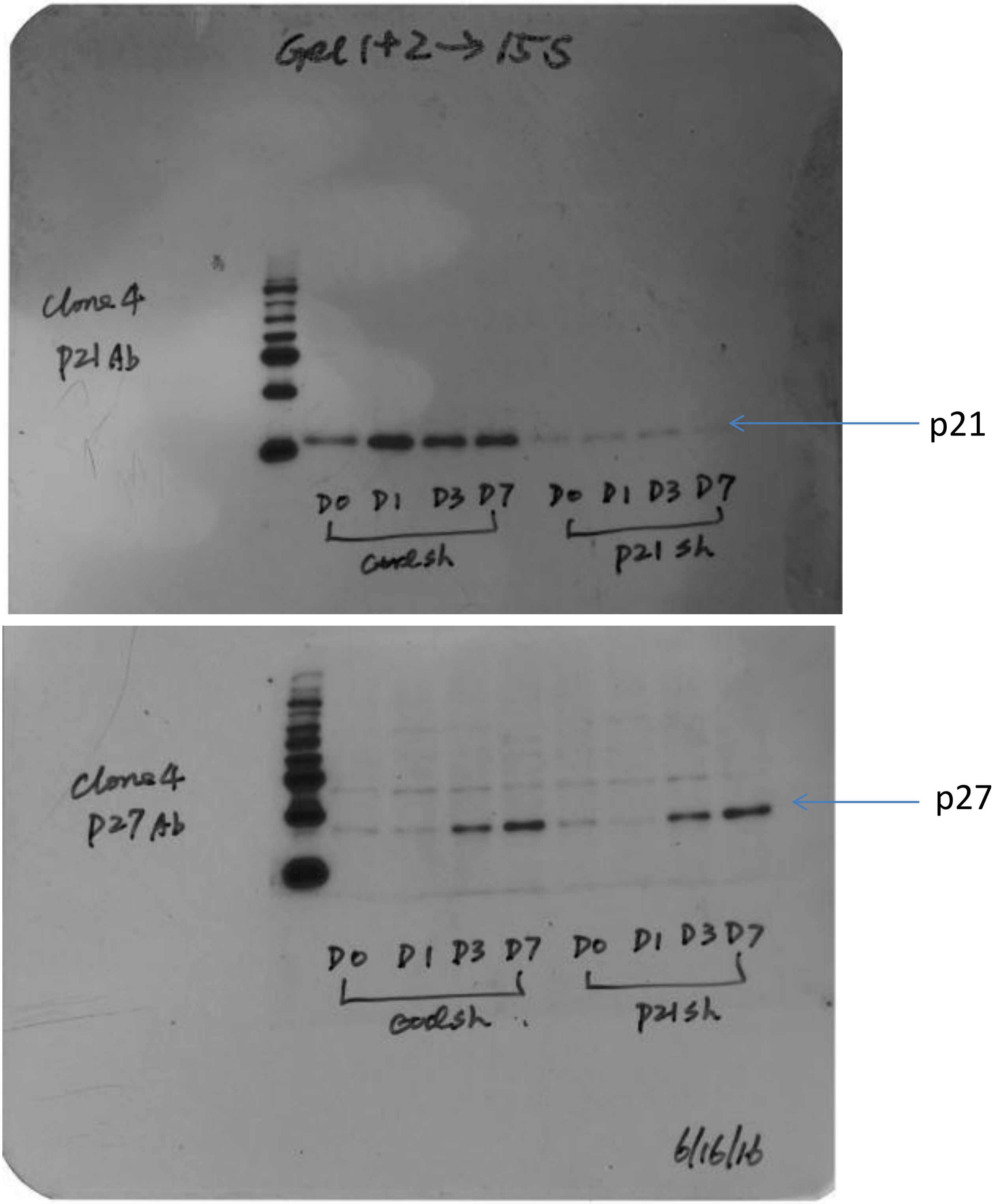

Figure 5B_Clone4_GAPDH

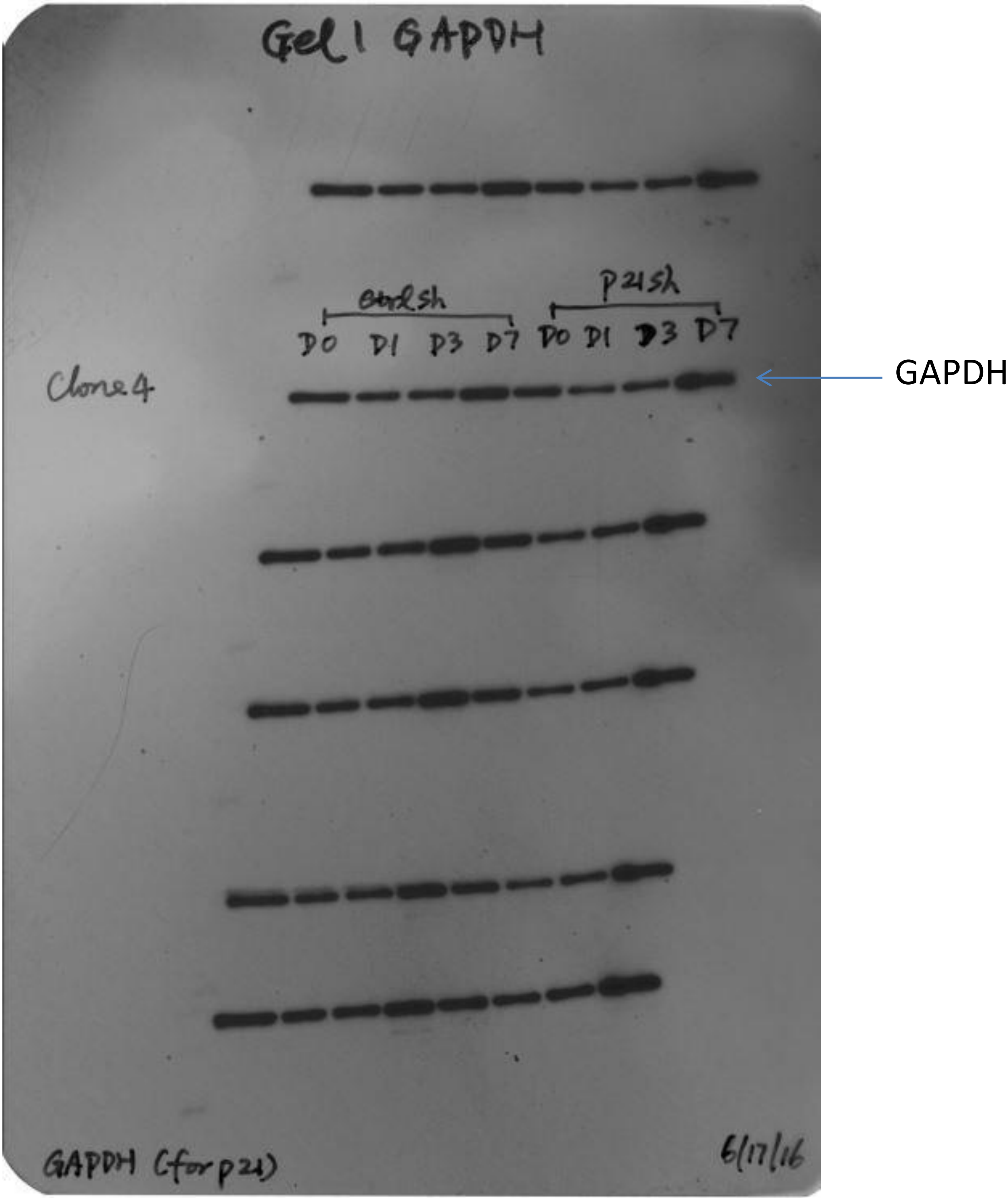

GAPDH

Figure 5D_Clone4_p27

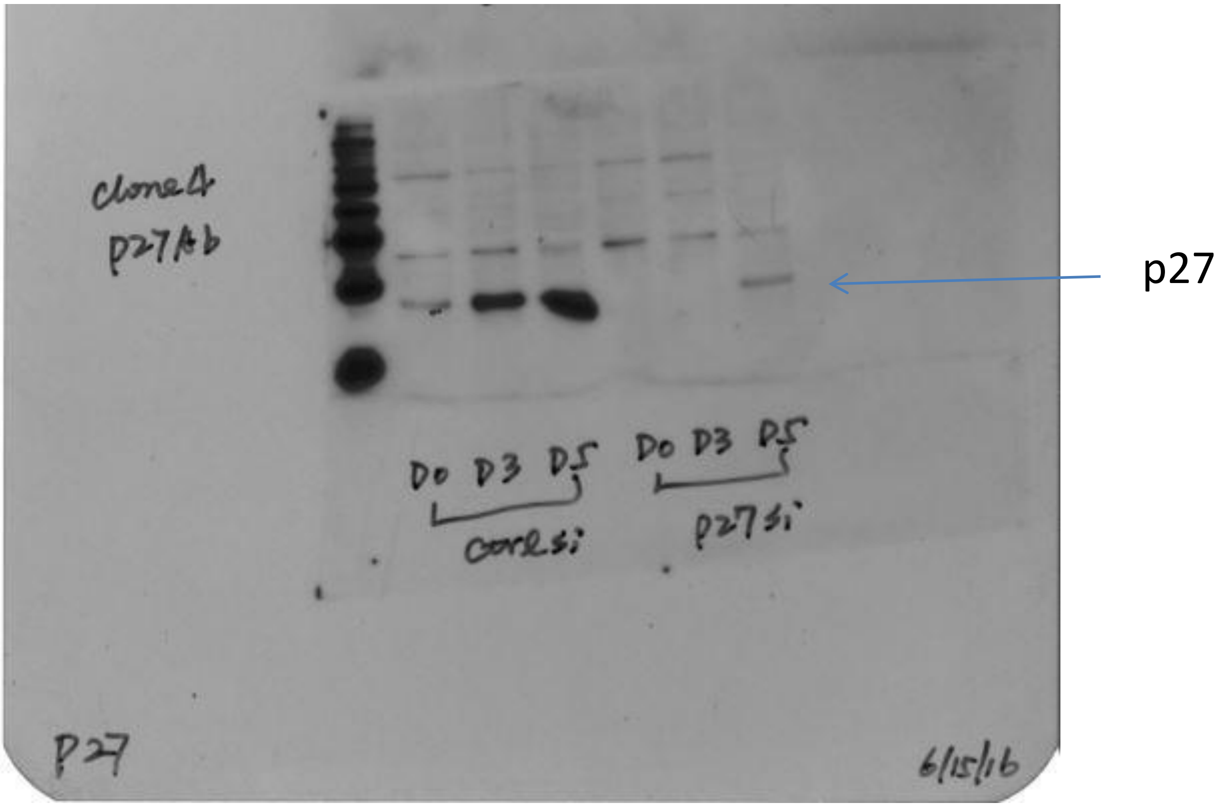

Figure 5D_Clone4_GAPDH

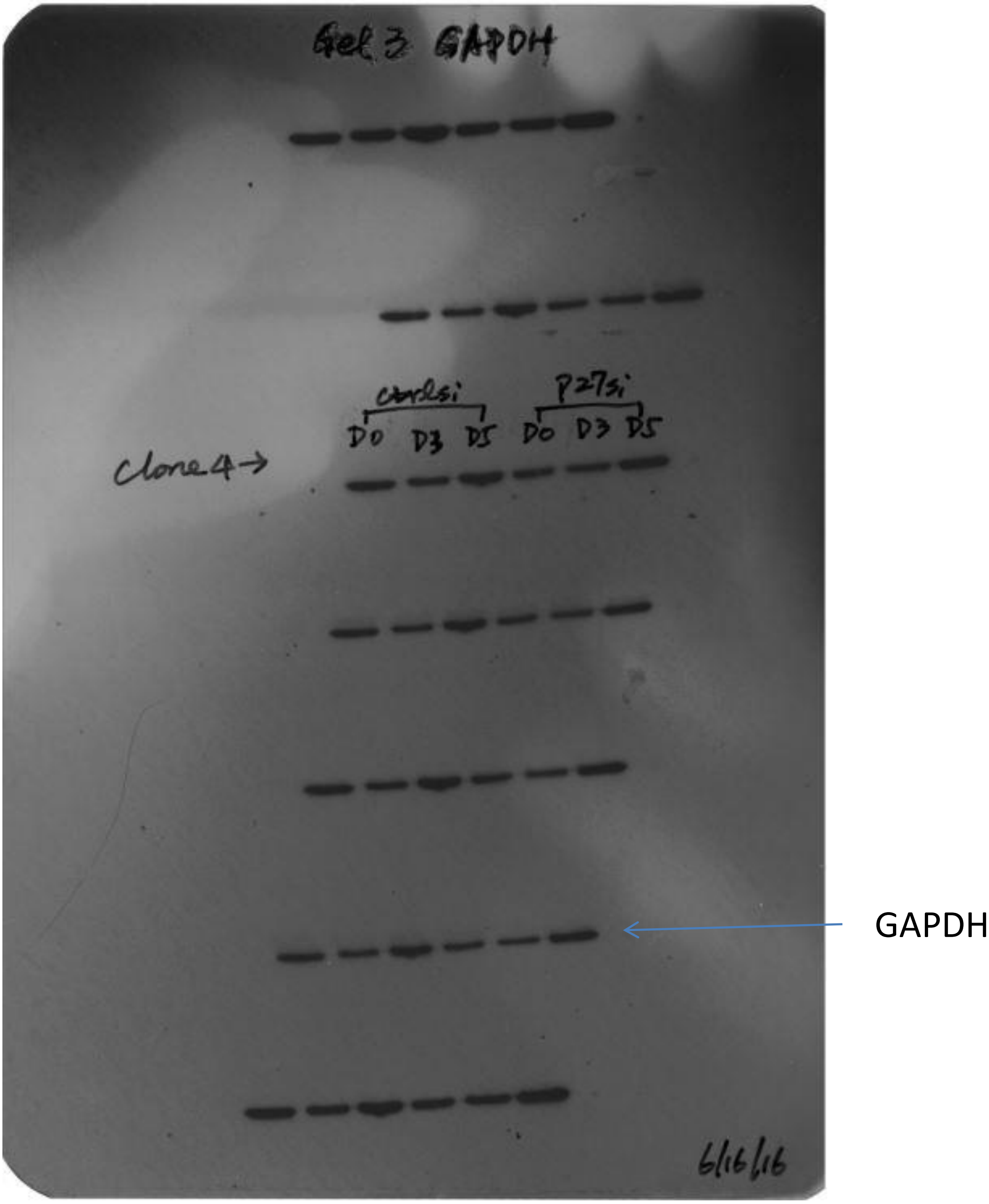

GAPDH

Figure 5G_Clone4_p27

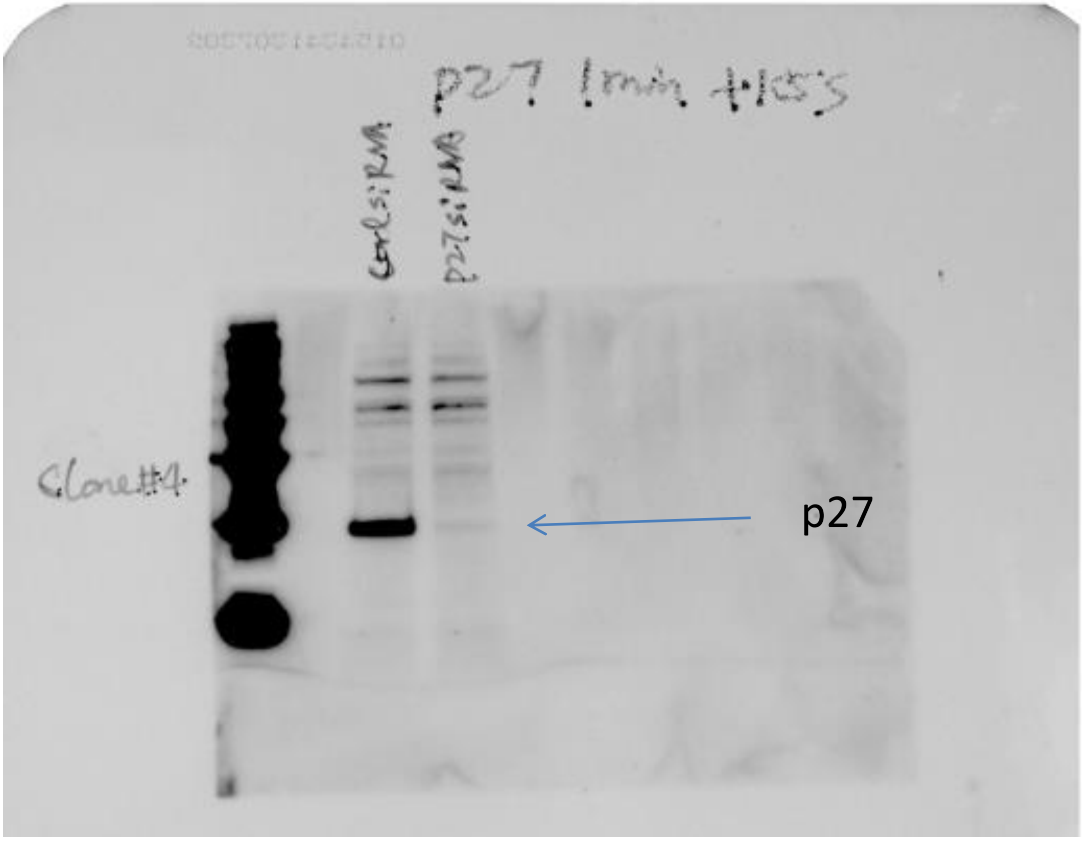

Figure 5G_Clone4_GAPDH

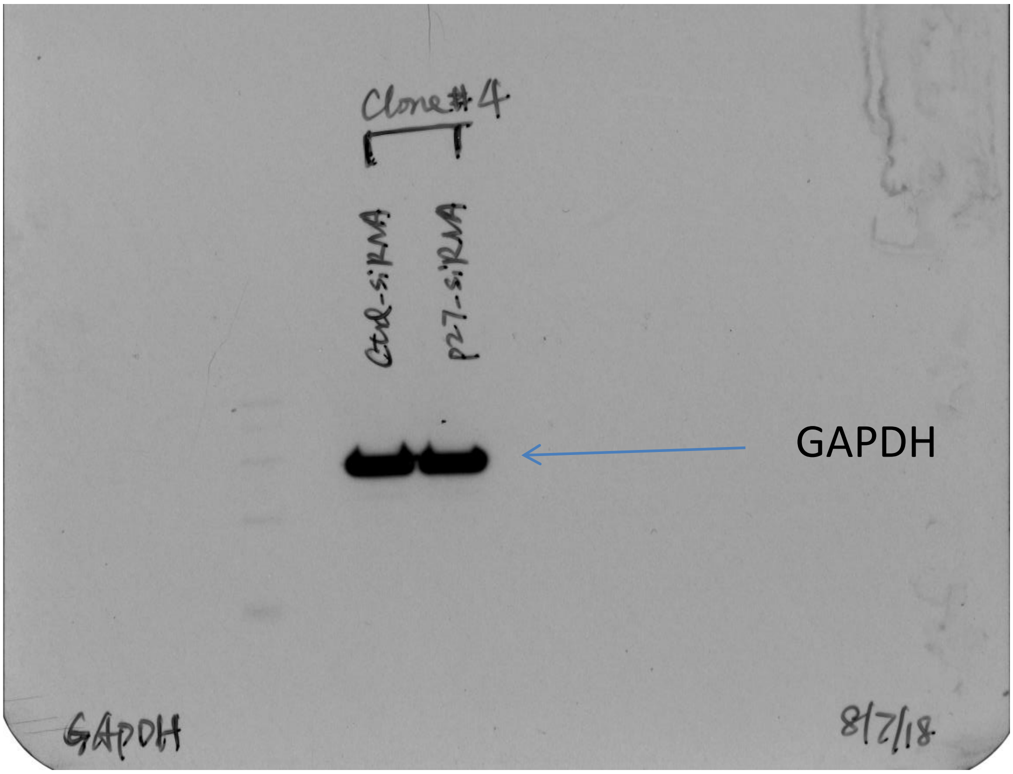

Figure 5H_Clone4_p27

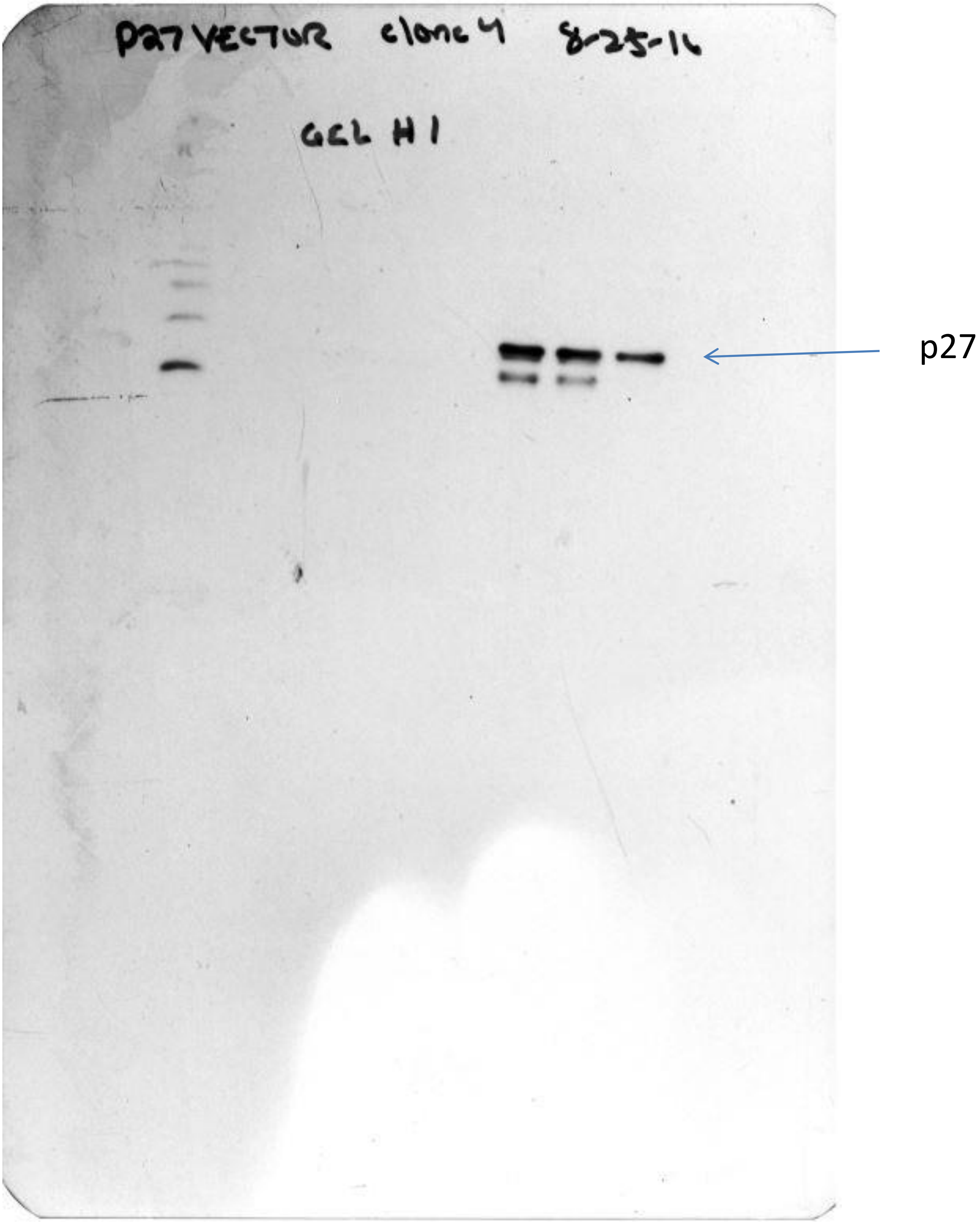

Figure 5H_Clone4_GAPDH

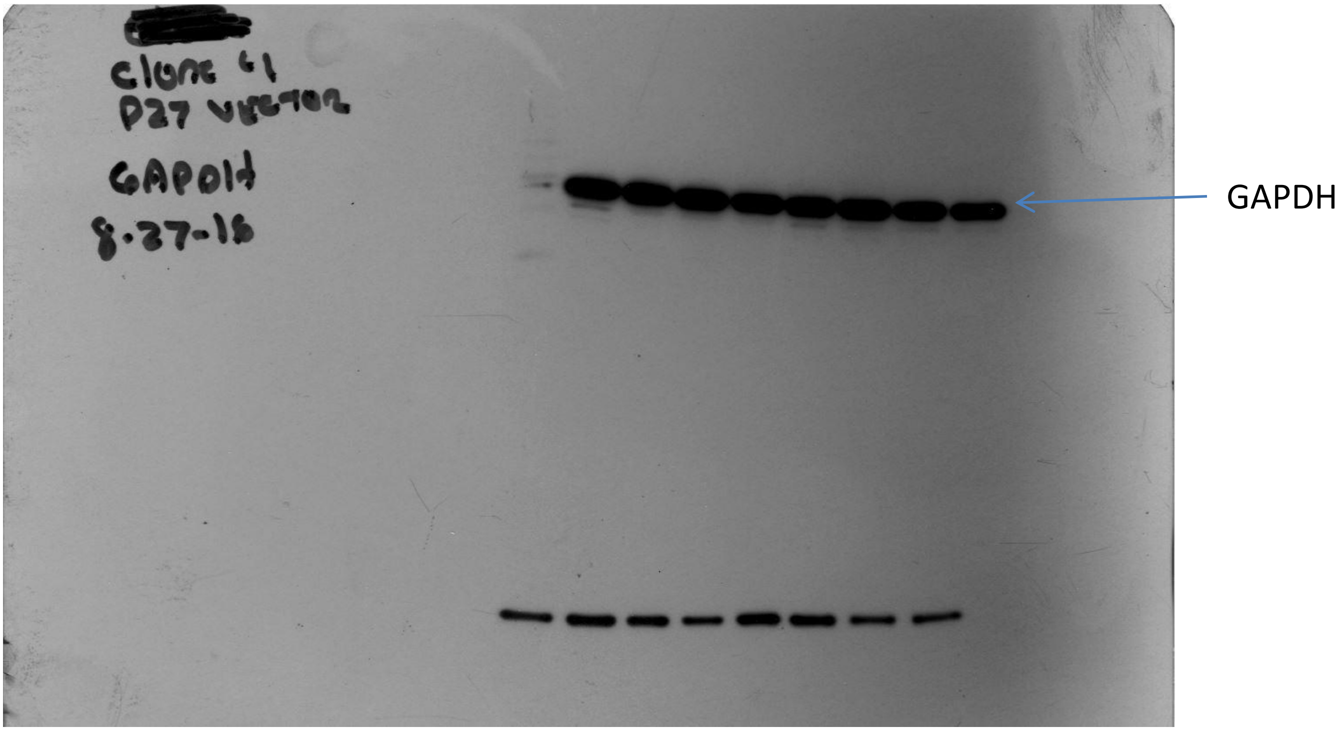

GAPDH

Figure 7A_Clone4_FOXO3_FOXO4

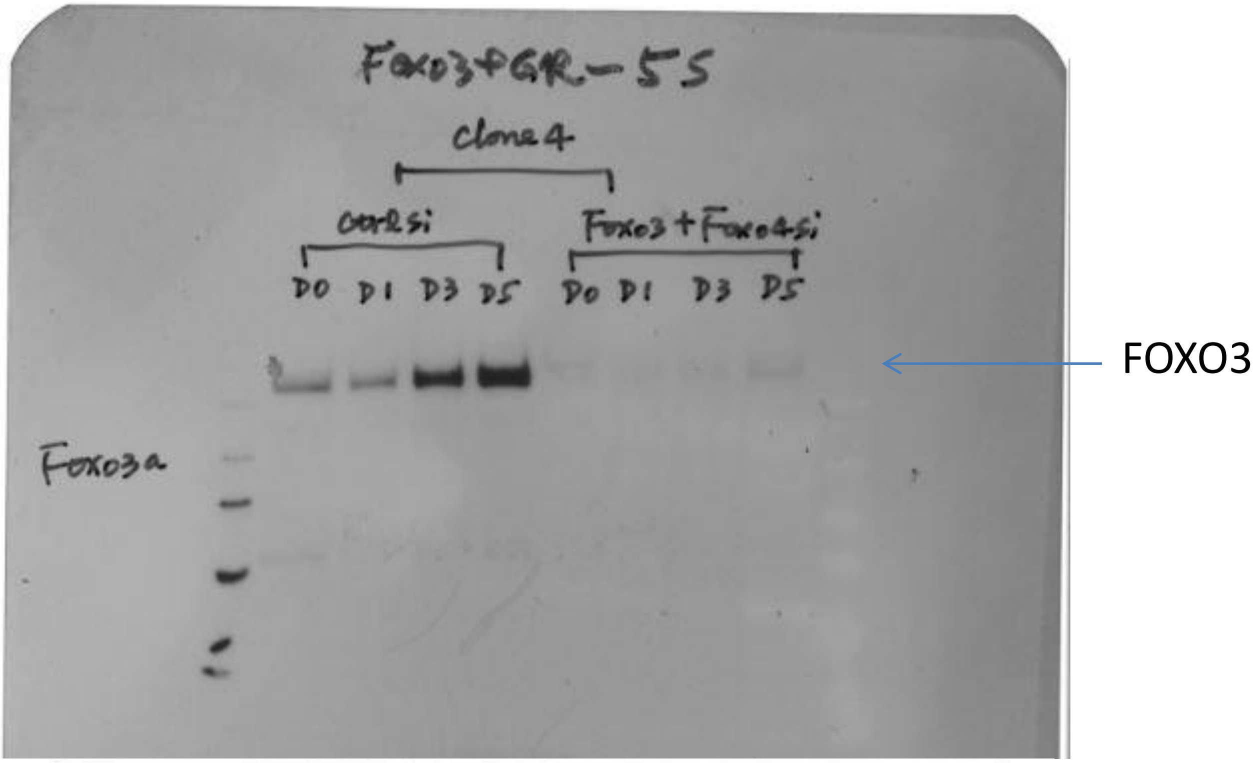

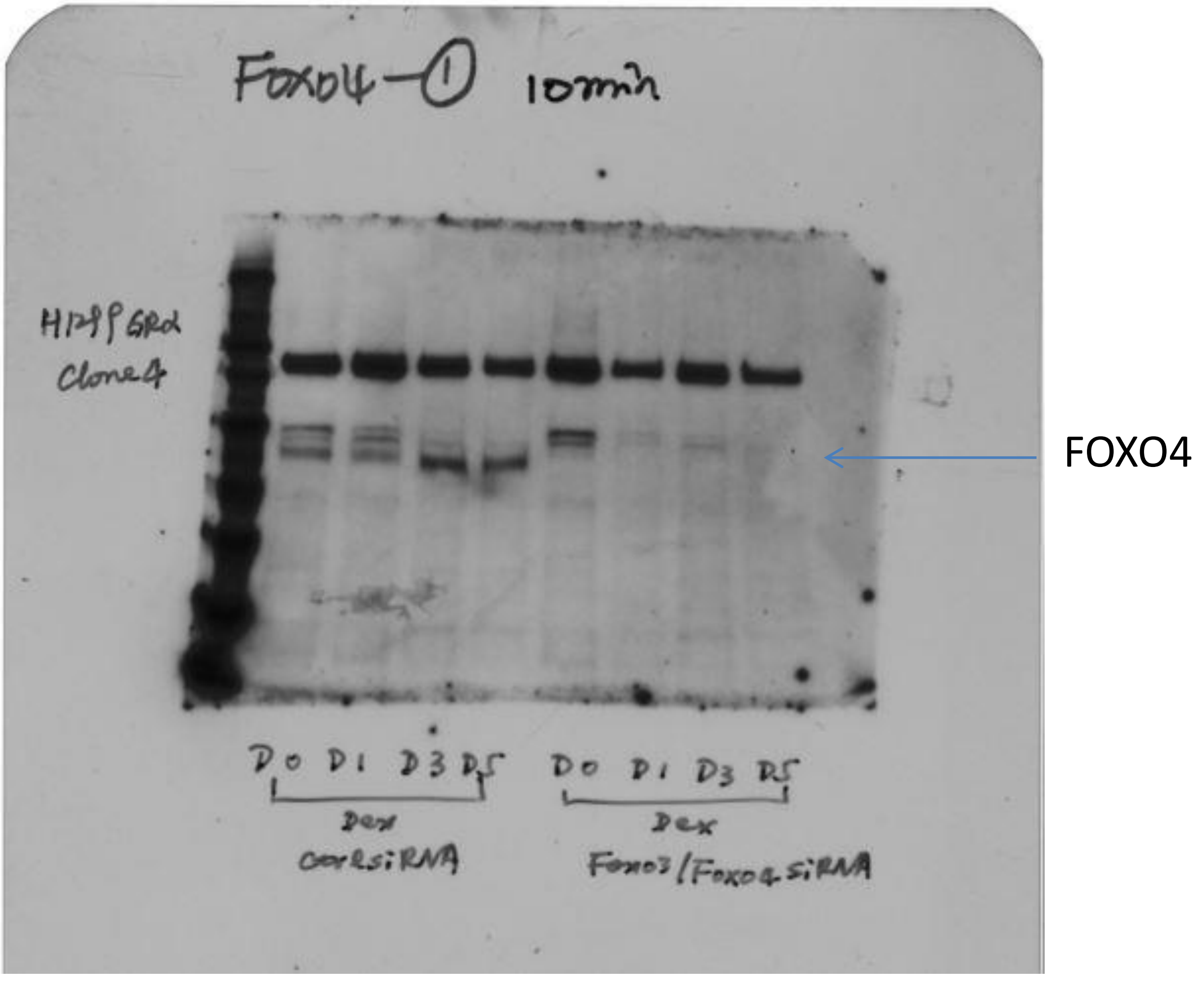

Figure 7A_Clone4_p27

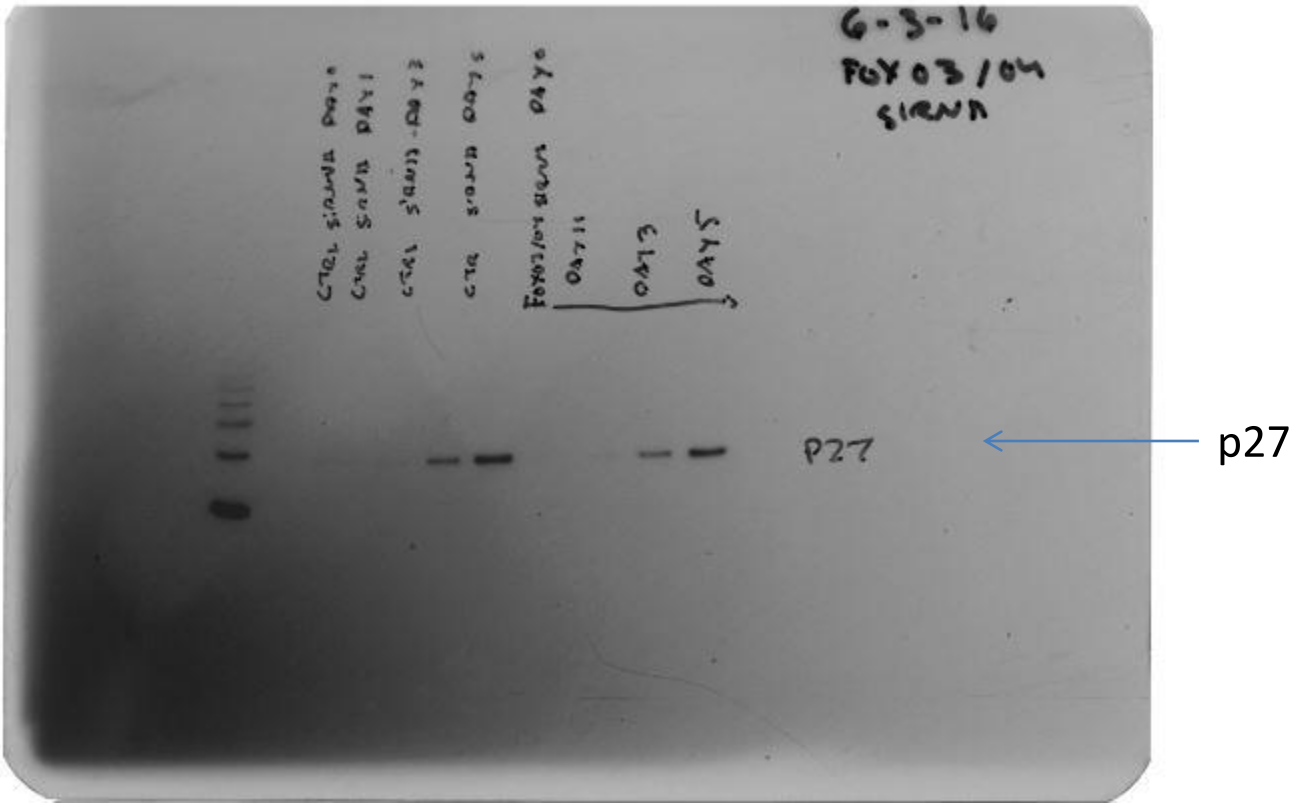

p27

Figure 7A_Clone4_GAPDH

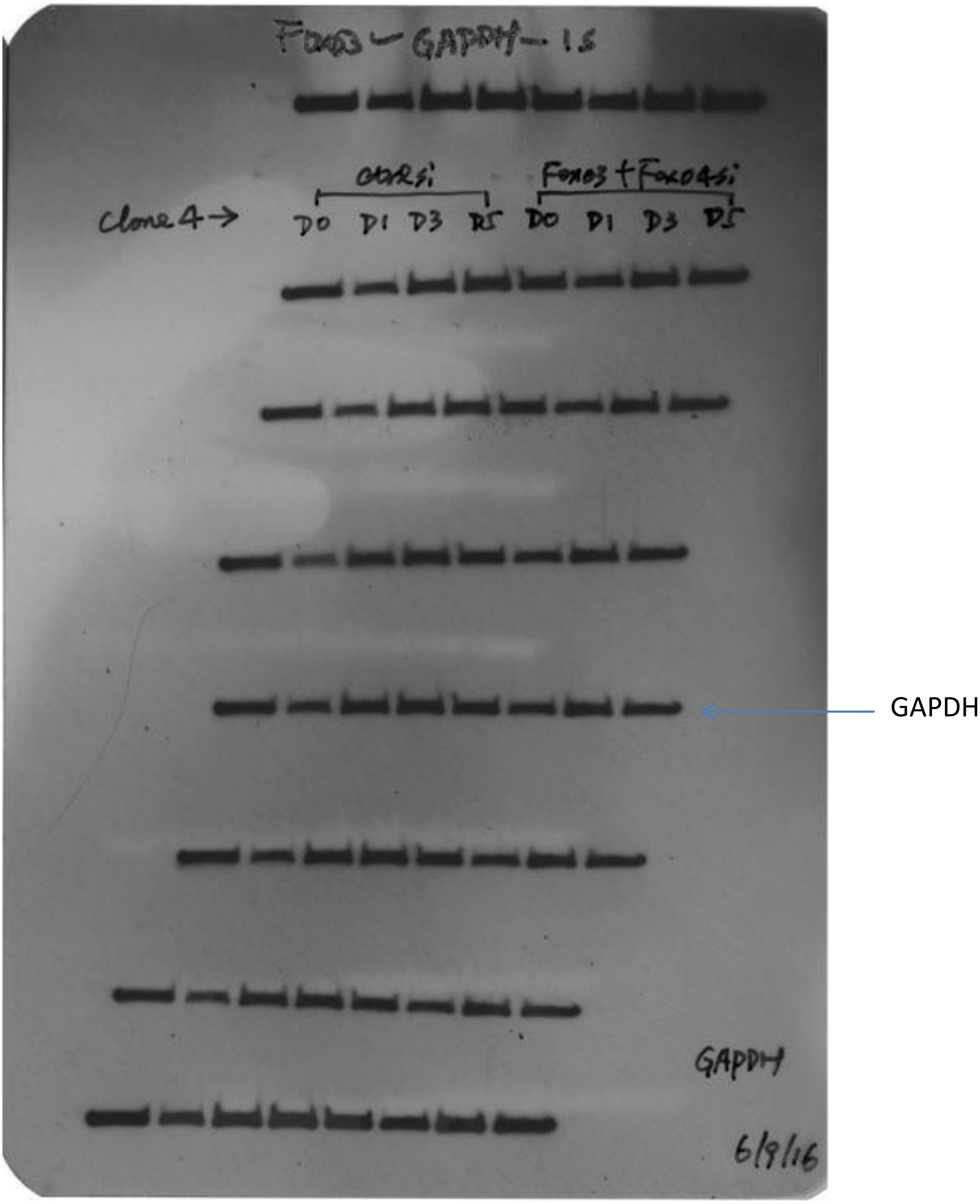

GAPDH

Figure 7D_Clone4_p21

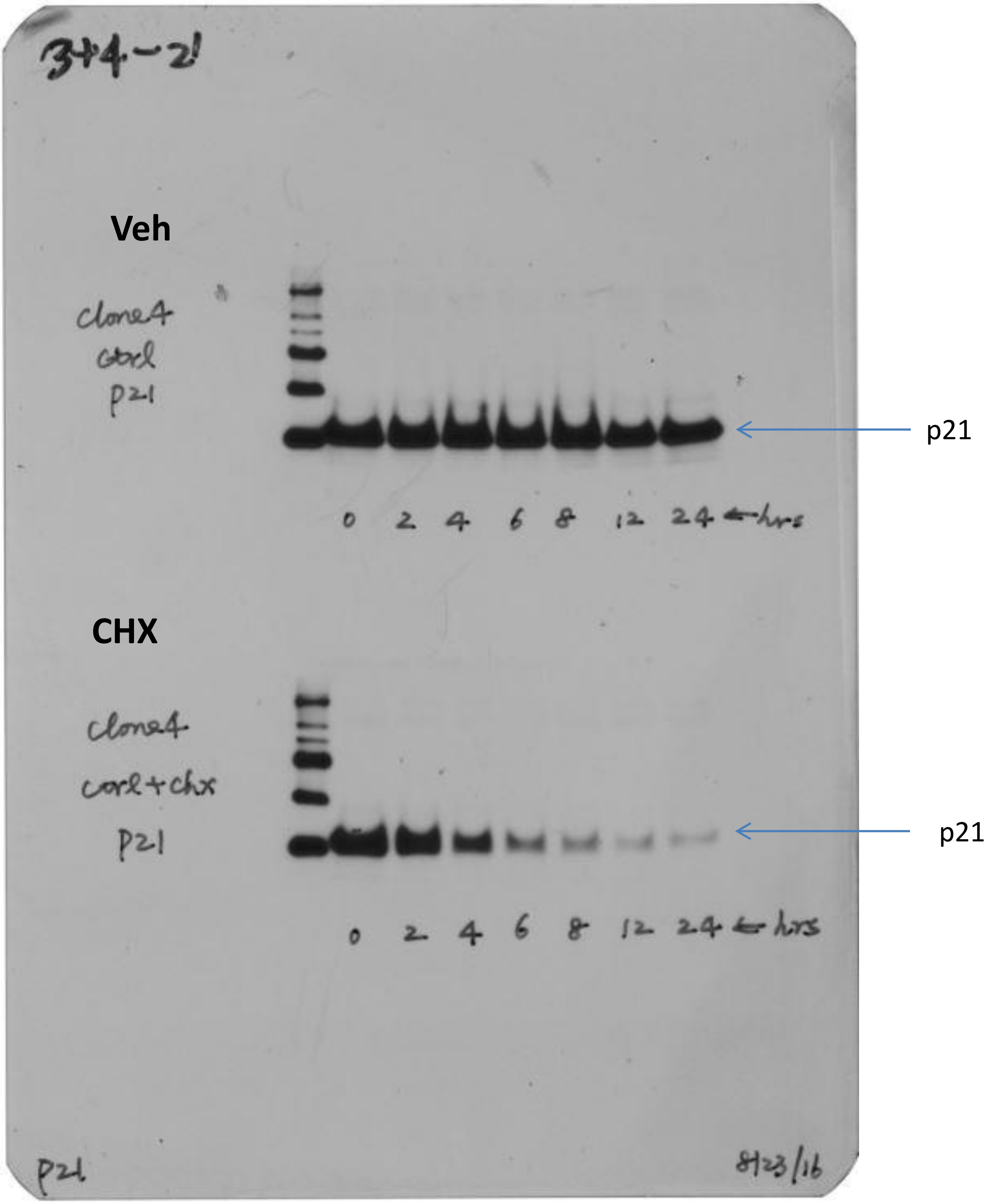

Figure 7E_Clone4_NoDex_p27

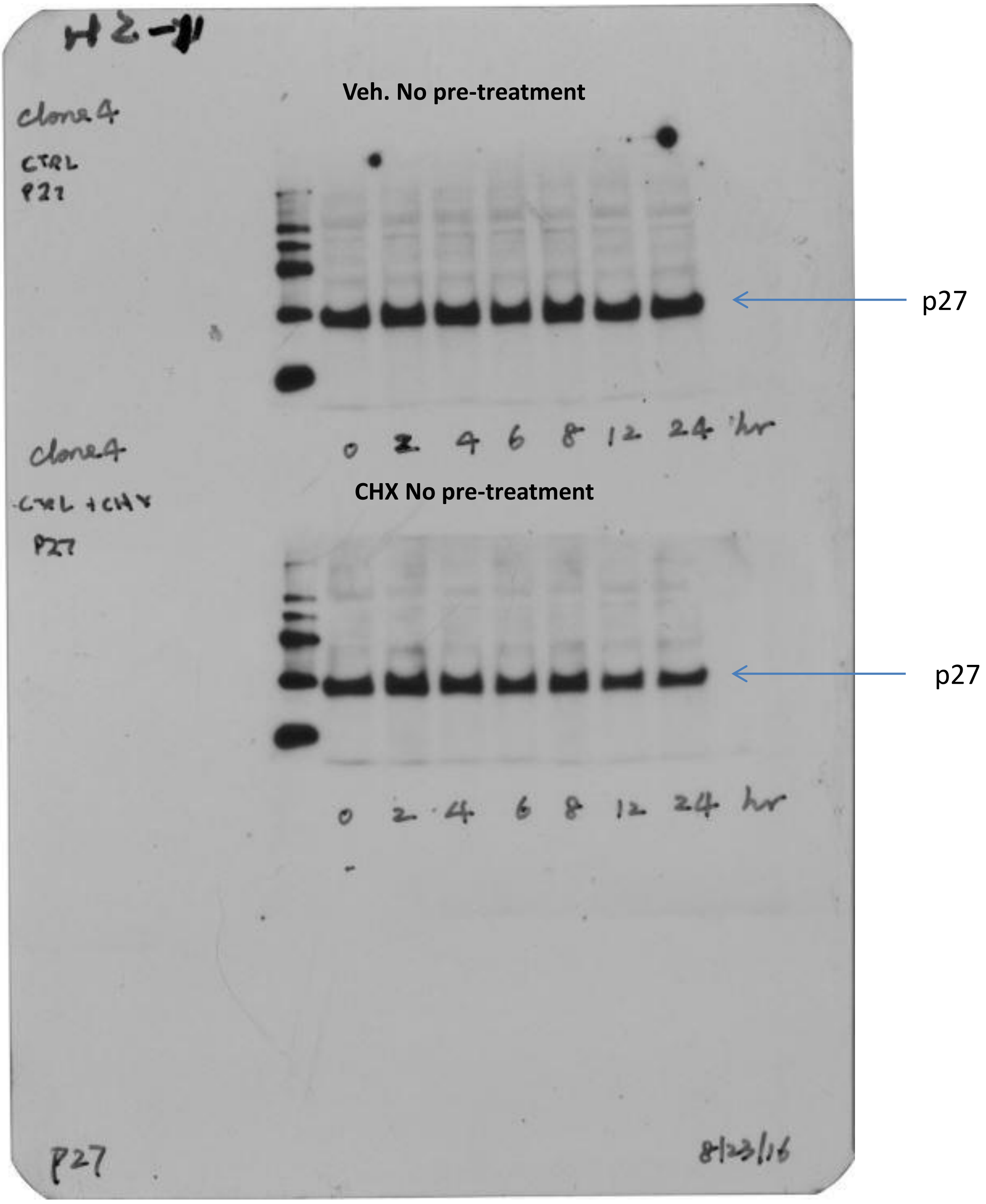

Figure 7E_Clone4_NoDex_α-Tubulin

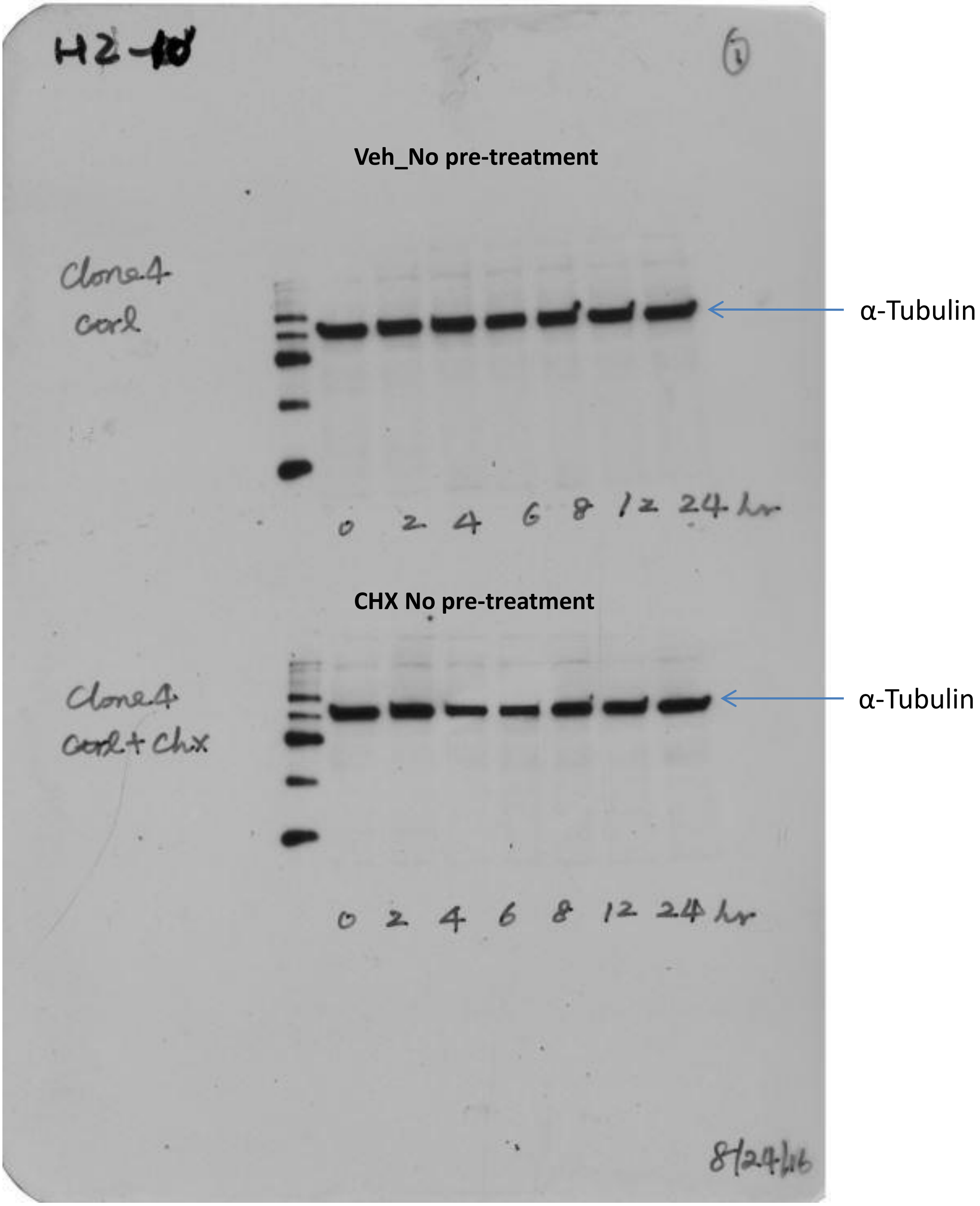

Figure 7E_Clone4_Dex_p27

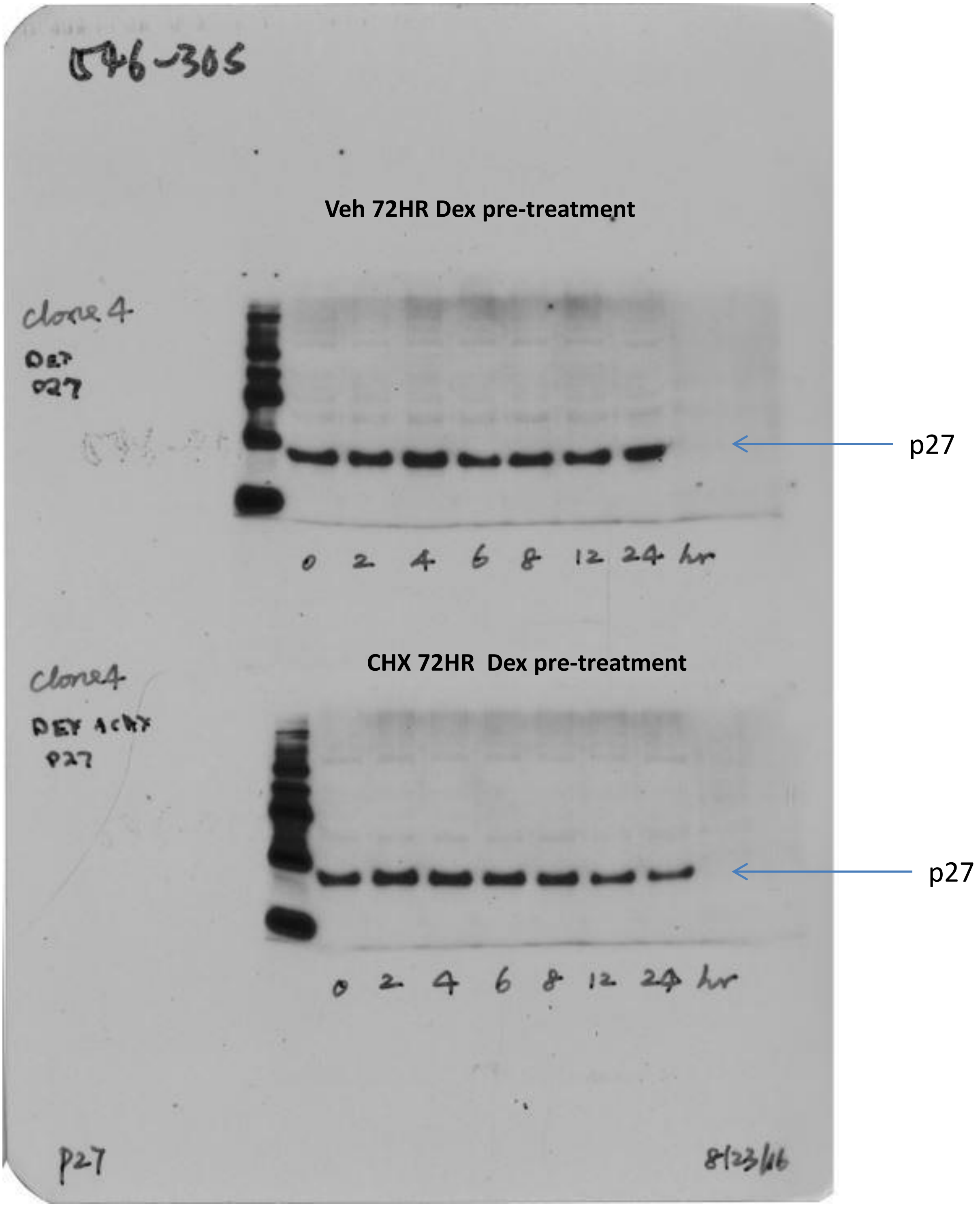

Figure 7E_Clone4_Dex_ α-Tubulin

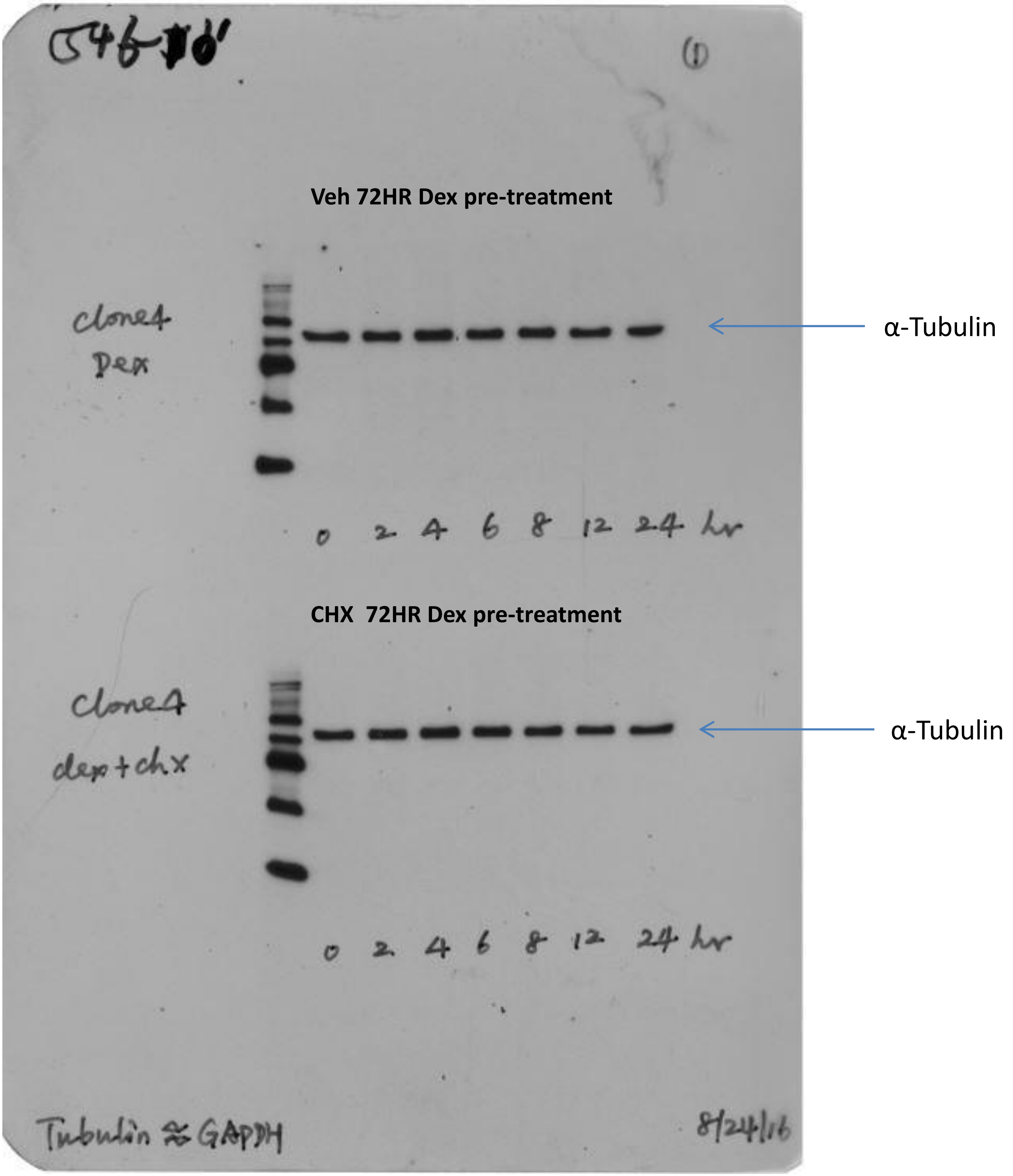

Supplement: Supplementary file 2 — Original Autorads [file 41598_2018_34475_MOESM2_ESM.pdf]
